# Supplementary material for: Genome-Wide Identification and Analysis of Lbd Transcription Factor Genes in Jatropha curcas and Related Species
Source: Plants (Basel). 2022 Sep 14;11(18):2397. doi: 10.3390/plants11182397 (PMC9504267; doi:10.3390/plants11182397)
Supplement: Supplementary file 1 [file plants-11-02397-s001.zip › plants-1876162-supplementary.pdf]

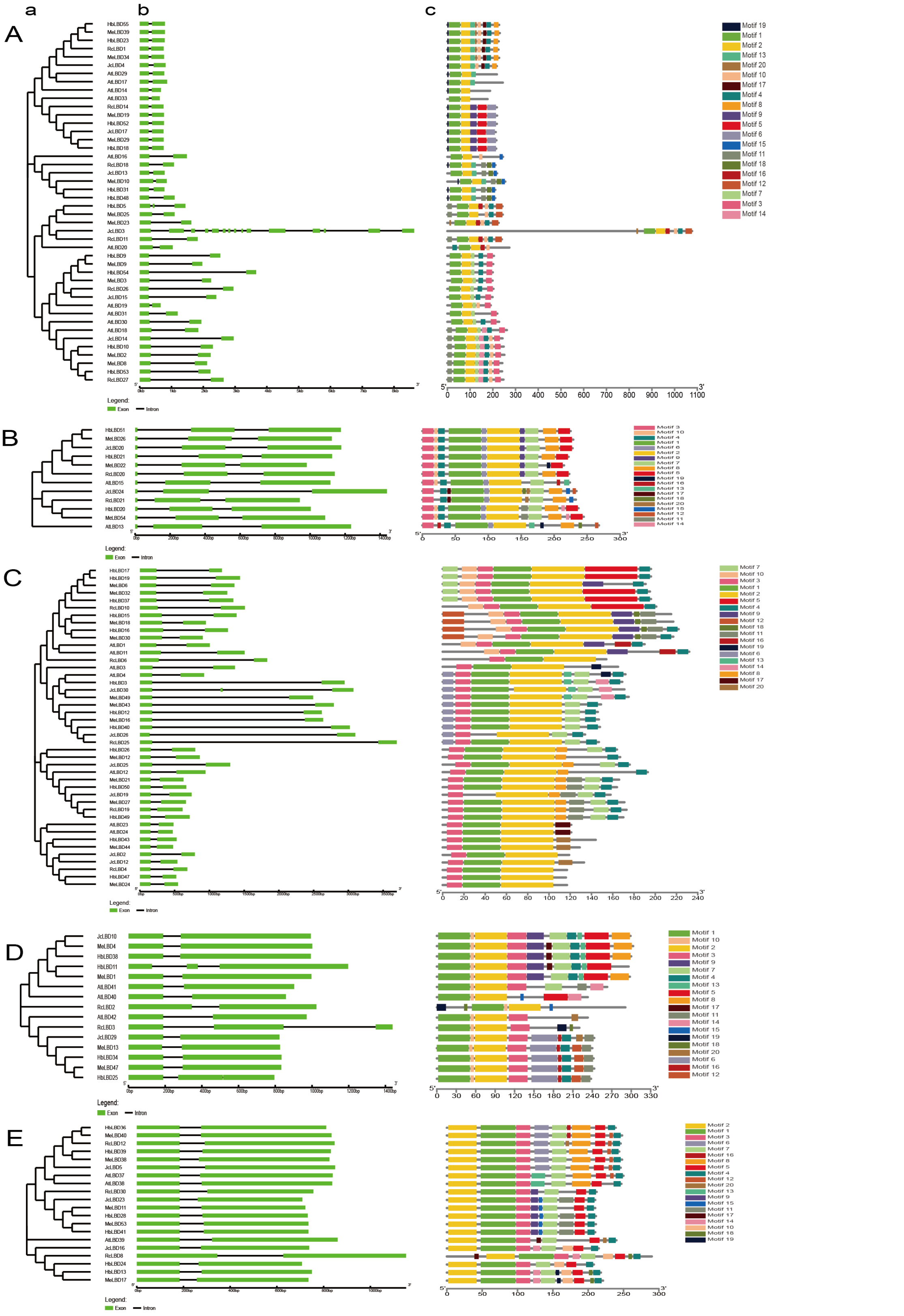

Figure S1. The phylogenetic relationship, gene structure, and conserved motifs of Euphorbiaceae LBD proteins. A, B, C, D, and E are phylogenetic tree, gene structure and conserved motif of Euphorbiaceae species *LBD*s grouped into Ib, Ic, Id, IIa and IIb, respectively.

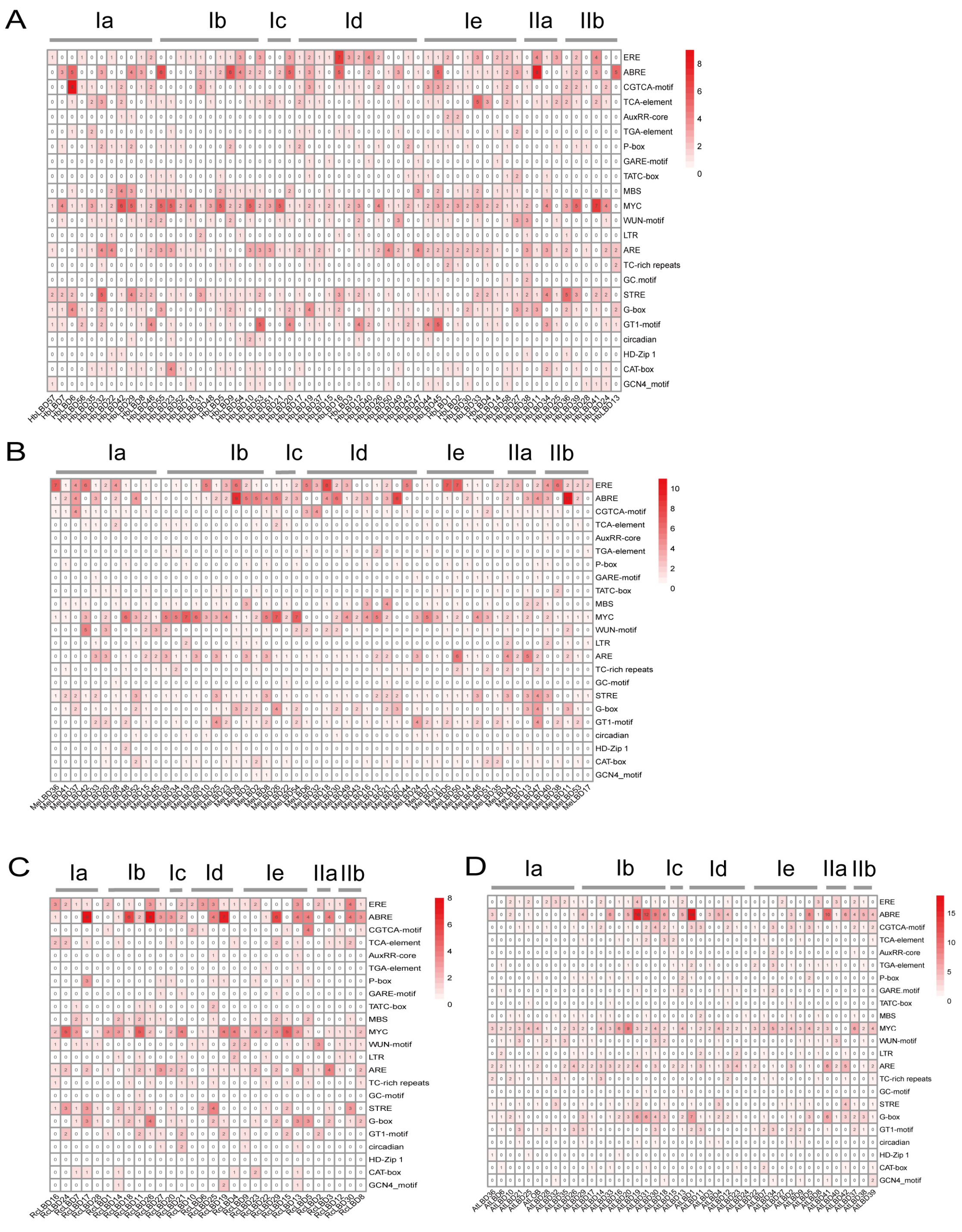

Figure S2. Cis-acting element matrinx identified in the 1500-bp upstream promoters of each *LBD* genes. A, B, C and D represent rubber tree, cassava, castor, and *A. thaliana*, respectively.

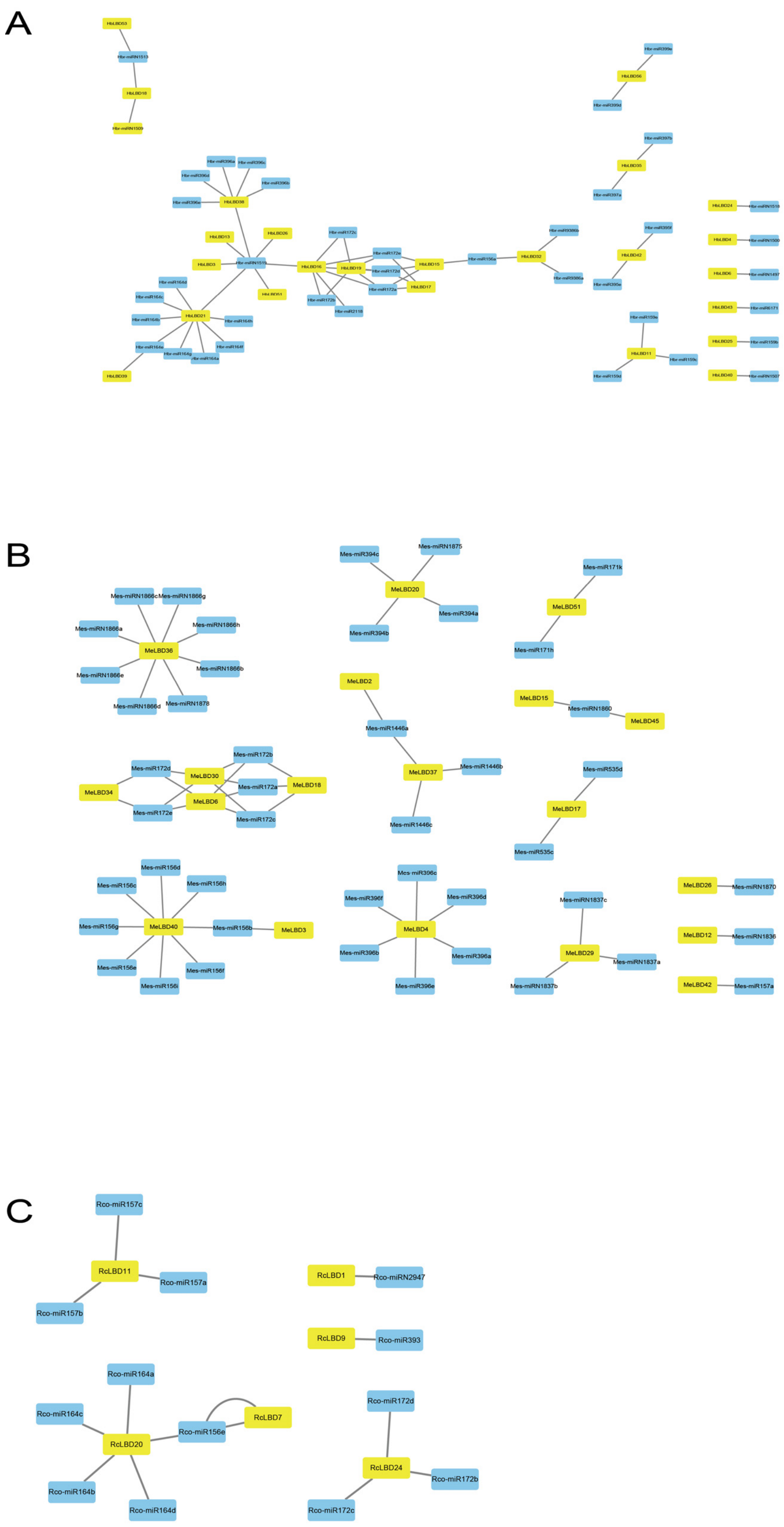

Figure S3. Netwrok between the miRNAs and their targeted *LBD* genes. The blue and yellow rectangles indicate miRNAs and target genes, respectively. A, B, and C represent rubber tree, cassava, and castor, respectively.

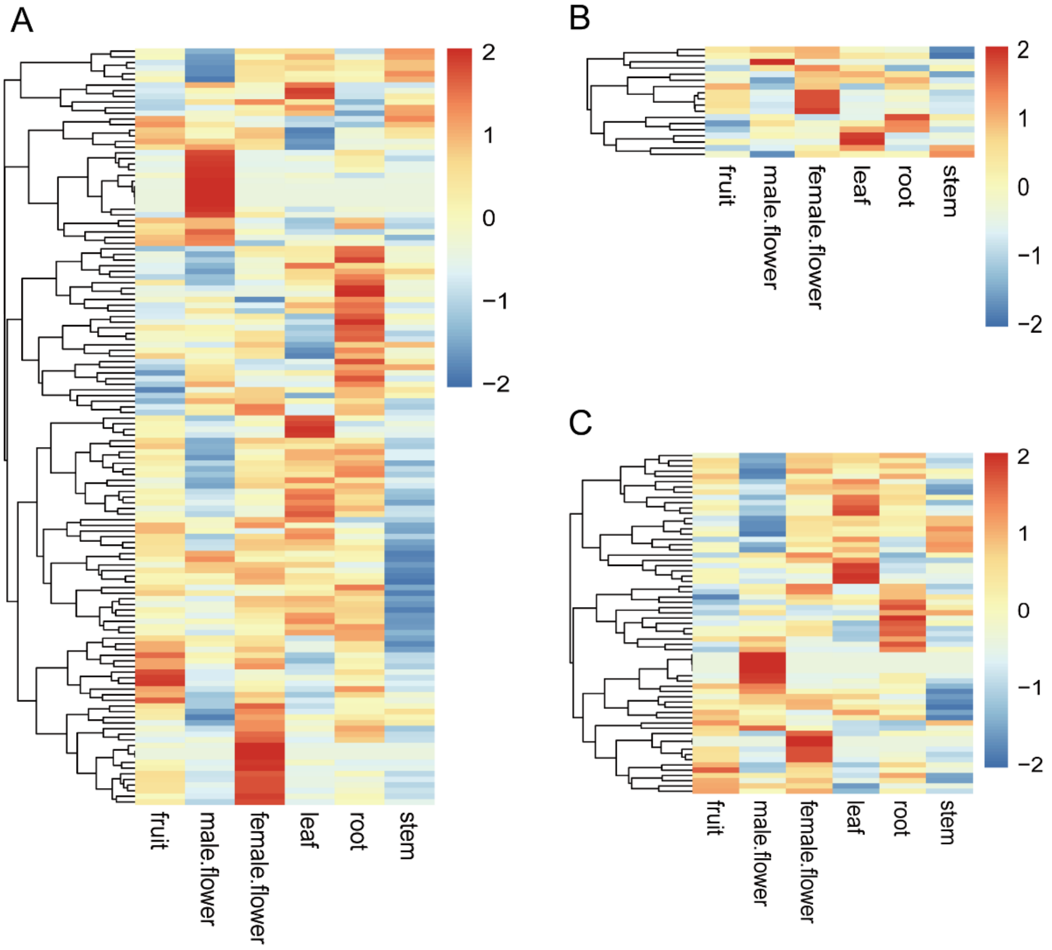

Figure S4. Tissue expression patterns of JcLBD target genes. A-C are the downstream target genes of JcLBD27, JcLBD24 and JcLBD22, respectively.

Table S1. Basic information of *LBD* genes of Euphorbiaceae

Gene Name	Protein ID	Gene ID	Chrom Name	Start	End	Strand	Size(aa)	MW	pI	A.I.	Instability	GRAVY
HbLBD1	XP_021684898.1	LOC110668103	NW_018745707.1	3646668	3648545	+	250	28786.52	5.24	87.36	58.23	-0.532
HbLBD2	XP_021684970.1	LOC110668150	NW_018745707.1	3694109	3696047	+	250	28787.5	5.15	87.36	59.77	-0.532
HbLBD3	XP_021639540.1	LOC110634723	NW_018745719.1	1470523	1473927	-	169	18424.02	8.6	77.87	62.86	-0.215
HbLBD4	XP_021665987.1	LOC110654342	NW_018745762.1	450010	452402	+	260	29575	6.58	67.96	59.13	-0.701
HbLBD5	XP_021667818.1	LOC110655692	NW_018745787.1	1770063	1771564	-	243	26647.88	5.81	75.1	66.58	-0.373
HbLBD6	XP_021668033.1	LOC110655868	NW_018745789.1	560019	563365	-	321	36151.52	6.64	63.27	74.38	-0.74
HbLBD7	XP_021668010.1	LOC110655852	NW_018745789.1	572879	573829	-	299	33464.61	6.66	69.57	57.36	-0.594
HbLBD8	XP_021668362.1	LOC110656096	NW_018745792.1	1662974	1665246	-	185	20387.97	6.81	60.22	63.94	-0.544
HbLBD9	XP_021668577.1	LOC110656244	NW_018745794.1	307999	311060	-	205	22215.33	8.71	86.15	46.89	-0.078
HbLBD10	XP_021668578.1	LOC110656245	NW_018745794.1	318613	321422	+	248	25589.79	8.14	70.04	76.95	-0.15
HbLBD11	XP_021675368.1	LOC110661113	NW_018745888.1	1297344	1298978	-	296	32023.5	8.33	84.02	44.1	-0.276
HbLBD12	XP_021676589.1	LOC110662022	NW_018745911.1	1527460	1530624	+	146	15973.13	6.88	75.48	54.7	-0.263
HbLBD13	XP_021677864.1	LOC110662995	NW_018745937.1	1455058	1456026	+	218	23750.18	5.76	81.83	53.9	-0.089
HbLBD14	XP_021679170.1	LOC110663972	NW_018745966.1	236175	237802	-	284	31980.44	5.59	66.69	58.41	-0.665
HbLBD15	XP_021679463.1	LOC110664196	NW_018745972.1	220196	222028	+	215	23499.76	5.92	76.79	72.88	-0.172
HbLBD16	XP_021680496.1	LOC110664918	NW_018745996.1	441735	443529	-	222	24010.31	5.6	78.29	83.3	-0.16
HbLBD17	XP_021681468.1	LOC110665577	NW_018746009.1	1278134	1279380	-	196	21374.16	5.35	71.33	79.23	-0.31
HbLBD18	XP_021683712.1	LOC110667224	NW_018746054.1	1140775	1145721	+	215	23665.52	5.21	78.56	51.21	-0.173
HbLBD19	XP_021683852.1	LOC110667349	NW_018746059.1	825445	827230	+	196	21400.3	5.35	74.23	77.72	-0.25
HbLBD20	XP_021684524.1	LOC110667867	NW_018746072.1	841327	842521	-	237	25776.26	8.61	77.38	80.93	-0.315
HbLBD21	XP_021684825.1	LOC110668075	NW_018746076.1	384192	386475	-	222	24143.53	9.02	71.22	76.68	-0.37
HbLBD22	XP_021685282.1	LOC110668385	NW_018746081.1	91134	91882	+	170	19111.76	8.21	74.65	70.07	-0.272
HbLBD23	XP_021686927.1	LOC110669531	NW_018746114.1	116820	118181	+	226	24645.44	5.94	61.81	40.72	-0.371
HbLBD24	XP_021690332.1	LOC110671988	NW_018746183.1	79523	80360	-	208	22808.76	5.84	72.21	44.32	-0.287
HbLBD25	XP_021690526.1	LOC110672142	NW_018746196.1	15221	16332	-	238	26211.05	6.51	84.79	45.66	-0.306
HbLBD26	XP_021690689.1	LOC110672267	NW_018746203.1	404808	405669	+	164	18434.03	8.78	73.72	51.01	-0.266

HbLBD27	XP_021691222.1	LOC110672686	NW_018746220.1	661453	662594	+	250	28190.69	8.84	61.2	48.18	-0.791
HbLBD28	XP_021691667.1	LOC110673020	NW_018746229.1	478353	479642	+	211	22890.2	9.22	79.53	54.32	-0.154
HbLBD29	XP_021691713.1	LOC110673039	NW_018746230.1	197610	199559	-	167	18605.9	6.88	63.17	51.39	-0.51
HbLBD30	XP_021635423.1	LOC110631776	NW_018746267.1	722034	725598	-	233	26012.7	6.39	64.08	64.92	-0.817
HbLBD31	XP_021635859.1	LOC110632061	NW_018746273.1	390164	391543	+	211	23593.84	8.76	73.08	62.31	-0.352
HbLBD32	XP_021636999.1	LOC110632930	NW_018746312.1	290693	294513	+	221	24312.25	8.25	68.14	51.63	-0.487
HbLBD33	XP_021637557.1	LOC110633316	NW_018746320.1	32591	34745	-	252	28063.83	8.35	75.95	47.12	-0.481
HbLBD34	XP_021637950.1	LOC110633592	NW_018746330.1	304711	305737	+	242	26723.28	6.38	79.01	50.8	-0.408
HbLBD35	XP_021638677.1	LOC110634087	NW_018746347.1	86529	90367	+	222	24284.18	8.25	70.5	48	-0.473
HbLBD36	XP_021639088.1	LOC110634412	NW_018746356.1	990	2281	-	239	26111.1	8.05	80.75	74.72	-0.168
HbLBD37	XP_021639460.1	LOC110634677	NW_018746367.1	14269	16018	-	196	21562.5	6.29	71.79	71.56	-0.317
HbLBD38	XP_021643936.1	LOC110637874	NW_018746503.1	192121	193513	-	300	32236.43	8.02	76.8	47.28	-0.38
HbLBD39	XP_021644428.1	LOC110638244	NW_018746516.1	106353	107823	+	244	26665.48	9.04	73.16	65.86	-0.355
HbLBD40	XP_021644716.1	LOC110638463	NW_018746526.1	35438	38980	-	148	16285.53	7.61	71.22	61.39	-0.33
HbLBD41	XP_021646781.1	LOC110639956	NW_018746600.1	275348	276426	-	210	23029.23	8.84	81.33	59.71	-0.177
HbLBD42	XP_021648653.1	LOC110641299	NW_018746669.1	27722	29700	-	165	18222.45	6.88	64.55	58.94	-0.509
HbLBD43	XP_021649066.1	LOC110641593	NW_018746686.1	216815	217404	+	144	16267.5	9.32	71.18	65.84	-0.49
HbLBD44	XP_021650016.1	LOC110642327	NW_018746730.1	39804	41178	+	315	35692.34	5.76	80.79	58.99	-0.534
HbLBD45	XP_021650001.1	LOC110642318	NW_018746730.1	49570	51198	+	313	35621.2	5.73	78.82	61.16	-0.577
HbLBD46	XP_021650387.1	LOC110642595	NW_018746749.1	148301	150605	-	186	20429.03	6.89	66.18	59.15	-0.473
HbLBD47	XP_021652563.1	LOC110644197	NW_018746860.1	128665	129730	+	116	13029.18	8.6	90.86	49.05	-0.024
HbLBD48	XP_021653516.1	LOC110644865	NW_018746906.1	27547	29479	-	211	23223.36	6.97	71.75	61.05	-0.241
HbLBD49	XP_021654436.1	LOC110645535	NW_018746964.1	174677	175779	-	170	18758.38	6.17	83.76	57.17	-0.163
HbLBD50	XP_021654459.1	LOC110645560	NW_018746965.1	123976	124716	-	164	18212.84	6.04	83.23	52.49	-0.166
HbLBD51	XP_021654778.1	LOC110645827	NW_018746988.1	57766	59623	-	225	24536.92	9.16	72.04	77.61	-0.428
HbLBD52	XP_021656006.1	LOC110646770	NW_018747096.1	97574	98458	-	218	24041.83	5.02	72.16	60.75	-0.269
HbLBD53	XP_021657344.1	LOC110647698	NW_018747222.1	4371	7032	-	241	24942.33	8.14	72.9	76.04	-0.111
HbLBD54	XP_021657345.1	LOC110647699	NW_018747222.1	18787	22893	+	200	21740.74	8.13	84.4	62.3	-0.142
HbLBD55	XP_021658319.1	LOC110648408	NW_018747354.1	66413	68831	+	228	25184.04	6.14	61.71	45.45	-0.362
HbLBD56	XP_021658599.1	LOC110648616	NW_018747390.1	57296	60688	-	309	34631.74	6.78	63.24	70.47	-0.708

HbLBD57	XP_021658595.1	LOC110648612	NW_018747390.1	66560	68353	-	333	37240.79	6.94	68.62	59.34	-0.606
HbLBD58	XP_021661230.1	LOC110650533	NW_018747913.1	21824	22731	-	216	24180.98	8.76	51.94	50.83	-0.877
JcLBD1	jc007534	jc007534	chr1	33092759	33093487	-	242	27679	5.12	83.8	54.27	-0.594
JcLBD2	jc000568	jc000568	chr2	4131390	4132181	+	119	13481.67	9.24	95.21	61.72	-0.079
JcLBD3	jc001994	jc001994	chr2	30086916	30095550	+	1077	117788.7	6.87	91.45	46.42	-0.044
JcLBD4	jc008273	jc008273	chr3	2337459	2338644	-	218	23926.56	5.45	61.83	47.58	-0.332
JcLBD5	jc009890	jc009890	chr3	29940371	29943213	+	246	27063.76	8.56	70.12	71.82	-0.435
JcLBD6	jc010057	jc010057	chr3	31123703	31124584	+	293	32769.48	5.91	67.65	68.11	-0.655
JcLBD7	jc010058	jc010058	chr3	31127455	31129932	+	282	31636.49	6.24	72.73	68.82	-0.631
JcLBD8	jc010059	jc010059	chr3	31137572	31138486	+	304	33925.88	6.36	61.38	62.92	-0.696
JcLBD9	jc010192	jc010192	chr3	31875853	31876899	-	245	27321.6	9.28	66.45	44.35	-0.806
JcLBD10	jc024015	jc024015	chr4	26189697	26191598	+	299	32606	8.87	81.54	43.75	-0.414
JcLBD11	jc002798	jc002798	chr5	958082	958963	+	293	32650.41	5.81	70.68	67.5	-0.577
JcLBD12	jc003946	jc003946	chr5	22873023	22874462	+	133	15016.07	8.72	67.59	56.73	-0.441
JcLBD13	jc004246	jc004246	chr5	24621899	24623215	-	219	23979.13	8.38	67.85	56.17	-0.309
JcLBD14	jc005177	jc005177	chr5	29703799	29708286	-	243	25165.58	8.51	75.93	76.66	-0.087
JcLBD15	jc005178	jc005178	chr5	29713870	29716968	+	199	21902.01	8.86	81.91	59.33	-0.219
JcLBD16	jc015544	jc015544	chr6	7003170	7004478	+	215	23196.36	6.59	80.74	67.1	-0.106
JcLBD17	jc016552	jc016552	chr6	28028951	28029975	+	213	23456.29	5.1	77.98	51.8	-0.185
JcLBD18	jc016732	jc016732	chr6	29813069	29813936	-	209	23791.03	7.57	76.12	72.49	-0.37
JcLBD19	jc016750	jc016750	chr6	29973563	29974466	+	158	17527.05	6.27	87.66	55.42	-0.108
JcLBD20	jc016797	jc016797	chr6	30233915	30236221	-	228	25007.42	9.01	68.51	77.29	-0.483
JcLBD21	jc013323	jc013323	chr8	4037152	4038926	+	260	29074.79	5.89	79.5	57.53	-0.455
JcLBD22	jc014622	jc014622	chr8	28532059	28532715	-	218	23937.89	8.25	69.54	49.54	-0.442
JcLBD23	jc017499	jc017499	chr9	3441377	3443171	-	210	22584.77	8.83	83.62	54.34	-0.11
JcLBD24	jc018856	jc018856	chr9	26534939	26536693	+	234	25895.48	8.78	76.71	79.43	-0.39
JcLBD25	jc018876	jc018876	chr9	26647471	26648772	-	176	19596.23	7.1	78.69	52.65	-0.307
JcLBD26	jc010498	jc010498	chr10	616328	619724	-	134	14684.61	6.27	71.34	64.73	-0.316
JcLBD27	jc011310	jc011310	chr10	4896873	4897430	+	185	20432.98	7.04	63.89	49.8	-0.496
JcLBD28	jc011472	jc011472	chr10	5815531	5816580	-	306	34189.04	5.84	71.14	56.05	-0.613

JcLBD29	jc011890	jc011890	chr10	8652887	8654313	-	243	26825.3	5.96	76.63	50.24	-0.447
JcLBD30	jc012433	jc012433	chr10	22647810	22651132	-	171	18575.35	8.96	81.05	49.93	-0.167
JcLBD31	jc021378	jc021378	chr11	1934384	1935851	+	332	37487.12	8.74	79.61	54.43	-0.651
JcLBD32	jc022184	jc022184	chr11	7030067	7030594	+	175	19381.57	5.79	63.09	51.96	-0.6
JcLBD33	jc022416	jc022416	chr11	9148990	9149775	-	211	24318.86	9.09	68.44	61.32	-0.694
MeLBD1	Manes.01G110000.1.p	Manes.01G110000	Chromosome01	23137090	23138516	-	297	32067.35	8.48	77.51	44.9	-0.374
MeLBD2	Manes.01G265300.1.p	Manes.01G265300	Chromosome01	33740451	33743054	-	250	25832.18	8.51	69.52	82.06	-0.17
MeLBD3	Manes.01G265400.1.p	Manes.01G265400	Chromosome01	33758823	33761456	+	198	21835.94	7.61	78.38	54.05	-0.158
MeLBD4	Manes.02G068400.1.p	Manes.02G068400	Chromosome02	5106630	5108019	-	302	32634.03	6.71	76.85	44.71	-0.326
MeLBD5	Manes.03G048400.1.p	Manes.03G048400	Chromosome03	4122624	4123494	-	261	29911.54	6.42	74.37	68.21	-0.635
MeLBD6	Manes.04G025300.1.p	Manes.04G025300	Chromosome04	2645577	2647259	-	190	20672.44	5.56	70.95	75.53	-0.221
MeLBD7	Manes.04G140100.1.p	Manes.04G140100	Chromosome04	26698259	26699014	-	251	28737.4	5.23	83.11	58.63	-0.506
MeLBD8	Manes.05G044500.1.p	Manes.05G044500	Chromosome05	3254262	3256710	-	241	24999.36	8.14	75.39	72.87	-0.11
MeLBD9	Manes.05G044600.1.p	Manes.05G044600	Chromosome05	3264974	3267144	+	201	22244.34	9	83.48	60.85	-0.254
MeLBD10	Manes.05G087800.1.p	Manes.05G087800	Chromosome05	6894800	6895806	+	254	28047.44	8.58	79.21	63.39	-0.059
MeLBD11	Manes.05G180800.1.p	Manes.05G180800	Chromosome05	24762145	24763119	-	209	22554.7	8.13	85.41	56.14	-0.035
MeLBD12	Manes.05G188800.1.p	Manes.05G188800	Chromosome05	26101305	26102167	-	166	18502.25	8.44	80.48	56.52	-0.166
MeLBD13	Manes.06G068500.1.p	Manes.06G068500	Chromosome06	18330826	18331652	+	239	26173.65	5.12	81.97	52.96	-0.331
MeLBD14	Manes.06G097500.1.p	Manes.06G097500	Chromosome06	20914462	20915524	+	294	32931.53	5.66	72.38	66.93	-0.586
MeLBD15	Manes.06G108700.1.p	Manes.06G108700	Chromosome06	21828155	21830418	-	185	20436.1	6.95	60.76	64.25	-0.542
MeLBD16	Manes.06G173400.1.p	Manes.06G173400	Chromosome06	27305151	27308303	+	146	16009.24	7.62	70.21	57.12	-0.314
MeLBD17	Manes.07G058400.1.p	Manes.07G058400	Chromosome07	6890438	6891502	+	220	23974.35	7.48	73.55	48.1	-0.24
MeLBD18	Manes.07G102200.1.p	Manes.07G102200	Chromosome07	23105287	23106645	+	216	23576.01	6.06	79.54	74.11	-0.134
MeLBD19	Manes.07G118600.1.p	Manes.07G118600	Chromosome07	24652426	24653392	+	218	24132.07	4.67	73.07	53.02	-0.225
MeLBD20	Manes.07G132200.1.p	Manes.07G132200	Chromosome07	25697536	25698858	-	171	18984.69	8.55	81.7	64.84	-0.161
MeLBD21	Manes.07G134700.1.p	Manes.07G134700	Chromosome07	25912090	25912774	+	165	18298.9	6.17	88.06	55.47	-0.16
MeLBD22	Manes.07G138500.1.p	Manes.07G138500	Chromosome07	26403736	26405477	+	214	23399.58	9.02	69.77	71.46	-0.384
MeLBD23	Manes.08G154100.1.p	Manes.08G154100	Chromosome08	31801958	31803707	+	225	24618.9	7.08	80.71	57.58	-0.211
MeLBD24	Manes.09G032700.1.p	Manes.09G032700	Chromosome09	4727734	4728283	-	116	13138.24	8.08	83.36	59.09	-0.166
MeLBD25	Manes.09G137400.1.p	Manes.09G137400	Chromosome09	25753012	25754109	+	243	26637.95	6.4	80.74	60.4	-0.332

MeLBD26	Manes.10G005700.1.p	Manes.10G005700	Chromosome10	441952	443794	+	228	24794.27	9.01	71.49	76.29	-0.393
MeLBD27	Manes.10G008800.1.p	Manes.10G008800	Chromosome10	680175	680863	-	170	18841.53	6.17	83.18	52.59	-0.126
MeLBD28	Manes.10G010200.1.p	Manes.10G010200	Chromosome10	794939	795796	+	171	19092.69	8.89	69.71	70.42	-0.317
MeLBD29	Manes.10G027400.1.p	Manes.10G027400	Chromosome10	2238554	2240468	-	215	23822.7	4.9	76.33	48.88	-0.22
MeLBD30	Manes.10G042900.1.p	Manes.10G042900	Chromosome10	3830274	3831489	+	216	23701.1	6.69	74.54	71.78	-0.208
MeLBD31	Manes.11G026000.1.p	Manes.11G026000	Chromosome11	2272404	2273147	+	247	28338.85	5.08	82.51	53.66	-0.617
MeLBD32	Manes.11G140800.1.p	Manes.11G140800	Chromosome11	25328208	25329869	+	194	21277.08	5.35	67.99	75.41	-0.279
MeLBD33	Manes.12G000100.1.p	Manes.12G000100	Chromosome12	20916	22841	+	221	24234.1	8.56	67.69	53.76	-0.523
MeLBD34	Manes.12G030700.1.p	Manes.12G030700	Chromosome12	2373012	2375258	-	227	24831.57	6.03	62.03	50.52	-0.4
MeLBD35	Manes.12G091800.1.p	Manes.12G091800	Chromosome12	13976847	13979498	-	257	28807.27	8.89	62.57	36.04	-0.79
MeLBD36	Manes.12G093700.1.p	Manes.12G093700	Chromosome12	14936620	14938922	+	300	33364.38	5.98	68.7	59.34	-0.606
MeLBD37	Manes.12G093800.1.p	Manes.12G093800	Chromosome12	14964472	14968053	+	302	33889.14	6.21	72.05	67.39	-0.612
MeLBD38	Manes.12G110600.1.p	Manes.12G110600	Chromosome12	25605195	25606435	+	247	27106.93	8.61	72.23	70.96	-0.366
MeLBD39	Manes.13G031900.1.p	Manes.13G031900	Chromosome13	2995261	2997536	-	230	25203.09	6.14	64.57	46.07	-0.355
MeLBD40	Manes.13G113000.1.p	Manes.13G113000	Chromosome13	23990699	23991927	+	247	27081.93	8.77	76.15	63.07	-0.364
MeLBD41	Manes.13G128100.1.p	Manes.13G128100	Chromosome13	25544935	25547012	+	299	33184.22	6.4	68.26	59.37	-0.535
MeLBD42	Manes.13G128200.1.p	Manes.13G128200	Chromosome13	25551631	25554253	+	287	32347.06	6.16	67.04	69.13	-0.721
MeLBD43	Manes.14G012200.1.p	Manes.14G012200	Chromosome14	1166656	1169919	-	148	16295.51	8.25	73.18	62.43	-0.322
MeLBD44	Manes.14G029300.1.p	Manes.14G029300	Chromosome14	2433536	2434013	-	128	14318.39	8.95	79.38	57.44	-0.279
MeLBD45	Manes.14G062100.1.p	Manes.14G062100	Chromosome14	5060059	5062273	+	185	20570.11	6.09	64.97	64.98	-0.524
MeLBD46	Manes.14G073600.1.p	Manes.14G073600	Chromosome14	5942407	5943402	-	290	32632.06	5.64	69.03	62.75	-0.673
MeLBD47	Manes.14G102500.1.p	Manes.14G102500	Chromosome14	8273112	8274407	-	242	26879.54	6.59	80.54	41.45	-0.376
MeLBD48	Manes.15G149900.1.p	Manes.15G149900	Chromosome15	11786336	11788445	-	172	18887.14	7.6	63.6	54.07	-0.488
MeLBD49	Manes.16G057100.1.p	Manes.16G057100	Chromosome16	16066664	16069337	+	174	19071.68	8.25	71.21	64.43	-0.307
MeLBD50	Manes.16G081100.1.p	Manes.16G081100	Chromosome16	23816043	23816684	-	213	23436.54	8.23	84.32	51.08	-0.391
MeLBD51	Manes.17G051500.1.p	Manes.17G051500	Chromosome17	18877608	18878366	+	252	28616.24	7.53	61.94	56.65	-0.796
MeLBD52	Manes.17G112700.1.p	Manes.17G112700	Chromosome17	25314973	25316856	+	179	19583.97	7.62	68.16	69.97	-0.502
MeLBD53	Manes.18G045200.1.p	Manes.18G045200	Chromosome18	3887324	3888368	-	210	22813.89	8.12	77.62	58.02	-0.16
MeLBD54	Manes.S010300.1.p	Manes.S010300	Scaffold00460	295160	296399	-	244	26469.02	7.69	74.75	84.91	-0.399
RcLBD1	XP_025015760.1	LOC8261015	NW_002995419.1	14056	15076	-	225	24675.26	5.94	57.78	46.78	-0.43

RcLBD2	XP_002533415.2	LOC8280381	NW_002995019.1	72098	73464	-	291	31918.76	9	91.89	40.92	-0.142
RcLBD3	XP_015582369.1	LOC8285784	NW_002994800.1	66782	68354	+	220	24407.28	9.45	87.32	58.58	-0.191
RcLBD4	XP_002530717.1	LOC8267077	NW_002994730.1	94459	95142	+	117	13033.05	8.83	86.84	59.15	-0.066
RcLBD5	XP_002530212.1	LOC8271164	NW_002994690.1	46022	46793	+	171	19270.11	5.84	85.56	63.34	-0.206
RcLBD6	XP_025014967.1	LOC8276720	NW_002994654.1	19764	21752	+	154	17057.45	8.81	80.52	78.99	-0.449
RcLBD7	XP_002528321.1	LOC8265382	NW_002994593.1	197486	198431	-	176	19514.24	8.55	80.45	63.82	-0.105
RcLBD8	XP_002527499.2	LOC8272260	NW_002994548.1	180593	182075	-	290	32238.03	8.64	80.69	58.62	-0.062
RcLBD9	XP_002527399.1	LOC8266956	NW_002994546.1	47689	48890	+	245	28021.51	4.97	88	65.11	-0.528
RcLBD10	XP_002526340.1	LOC8285998	NW_002994502.1	43823	45806	+	201	21871.9	4.86	77.31	77.3	-0.136
RcLBD11	XP_002525698.1	LOC8286506	NW_002994473.1	203877	206954	+	240	26129.36	6.07	81.38	70.11	-0.279
RcLBD12	XP_002525255.1	LOC8283348	NW_002994460.1	42360	43774	-	246	26988.67	8.91	68.17	69.4	-0.451
RcLBD13	XP_002525150.1	LOC8264235	NW_002994458.1	352749	354823	-	258	28775.22	8.95	57.79	38.72	-0.793
RcLBD14	XP_002524207.1	LOC8274859	NW_002994437.1	68898	69655	+	218	24370.34	4.53	75.69	48.28	-0.263
RcLBD15	XP_015576883.1	LOC8259176	NW_002994399.1	126632	128127	+	280	31373.07	9.07	70.71	56.06	-0.73
RcLBD16	XP_025013725.1	LOC8267241	NW_002994396.1	169842	173255	+	310	34490.75	6.4	72.16	65.44	-0.63
RcLBD17	XP_002521459.1	LOC8281783	NW_002994381.1	400912	402828	-	175	19271.66	7.63	61.37	62.89	-0.522
RcLBD18	XP_002521022.1	LOC8274770	NW_002994370.1	625165	626614	+	212	23248.35	7.53	73.25	62.51	-0.203
RcLBD19	XP_002520780.1	LOC8272084	NW_002994368.1	57754	58516	+	173	19274.92	6.21	85.14	54.05	-0.206
RcLBD20	XP_002520837.1	LOC8259521	NW_002994368.1	381740	383568	-	223	24321.76	9.01	71.79	73.07	-0.374
RcLBD21	XP_002517720.1	LOC8282744	NW_002994324.1	430457	432104	+	233	25336.73	8.21	64.55	69.88	-0.4
RcLBD22	XP_002517449.3	LOC8276004	NW_002994321.1	683342	683843	-	161	18110.76	8.88	76.96	54.6	-0.475
RcLBD23	XP_002516432.1	LOC8259574	NW_002994311.1	48398	49431	+	191	21088.64	6.88	71.1	75.11	-0.519
RcLBD24	XP_002514766.1	LOC8263444	NW_002994293.1	27292	30817	+	218	23763.77	8.24	70.46	47.82	-0.369
RcLBD25	XP_002513395.1	LOC8272614	NW_002994285.1	600899	605086	-	147	16099.32	7.61	69.73	60.35	-0.282
RcLBD26	XP_002511729.1	LOC8270647	NW_002994279.1	854816	858313	-	203	22322.4	8.72	78.33	58.94	-0.242
RcLBD27	XP_002511730.1	LOC8270648	NW_002994279.1	866075	869447	+	247	25434.91	8.4	76.28	71.53	-0.052
RcLBD28	XP_002511341.1	LOC8278307	NW_002994278.1	3151751	3153575	+	188	20110.76	6.29	61.86	62.82	-0.397
RcLBD29	XP_002511502.1	LOC8258162	NW_002994278.1	4227330	4228516	-	284	32024.58	5.79	66.34	68.4	-0.638
RcLBD30	XP_002510109.1	LOC8288504	NW_002994277.1	836487	837918	+	212	23301.7	8.62	80.94	49.71	-0.14

Table S2. Prediction of microRNA target sites for *LBD* genes in Euphorbiaceae

miRNA_Acc.	Target_ Acc.	Expectation	miRNA_ start	miRNA_ end	Target_ start	Target_ end	miRNA_ aligned_fragment	alignment	Target_ aligned_fragment	Inhibition	Multiplicity
Hbr-miR164e	HbLBD21	4	1	21	1271	1291	UGGAGAAGCAGGGCAC AUGCU	.:::: .:.: :::::: :	GAUAUGUUUCUUUCUU CUCCA	Cleavage	1
Hbr-miR172a	HbLBD16	4	1	21	448	468	AGAAUCUUGAUGAUGC UGCAU	::: ::::::::: : : :	CAGCAACAUCAUCAAG UUCCU	Cleavage	1
Hbr-miR172a	HbLBD19	4	1	21	296	315	AGAAUCUUGAUGAUGC UGCAU	::: ::::::::: ::: :	UAGCAACAUCAUCAAG- UUCU	Cleavage	1
Hbr-miR172b	HbLBD16	4	1	21	448	467	GGAAUCUUGAUGAUGC UGCAG	: ::: ::::::::: : :::	CAGCAACAUCAUCAAG- UUCC	Cleavage	1
Hbr-miR172c	HbLBD16	4	1	21	448	467	GGAAUCUUGAUGAUGC UGCAG	: ::: ::::::::: : :::	CAGCAACAUCAUCAAG- UUCC	Cleavage	1
Hbr-miR172d	HbLBD16	4	1	21	448	468	AGAAUCUUGAUGAUGC UGCAU	::: ::::::::: : : :	CAGCAACAUCAUCAAG UUCCU	Cleavage	1
Hbr-miR172d	HbLBD19	4	1	21	296	315	AGAAUCUUGAUGAUGC UGCAU	::: ::::::::: ::: :	UAGCAACAUCAUCAAG- UUCU	Cleavage	1
Hbr-miR172e	HbLBD16	4	1	21	448	468	AGAAUCUUGAUGAUGC UGCAU	::: ::::::::: : : :	CAGCAACAUCAUCAAG UUCCU	Cleavage	1
Hbr-miR172e	HbLBD19	4	1	21	296	315	AGAAUCUUGAUGAUGC UGCAU	::: ::::::::: ::: :	UAGCAACAUCAUCAAG- UUCU	Cleavage	1
Hbr-miR2118	HbLBD16	4	1	21	1015	1035	GAAAUGGGUGGAUGGG AGUGA	.:::. .::.::..:::: :.	CUACUUGUAUUCAUUC AUUUU	Cleavage	1
Hbr-miR9386a	HbLBD32	4	1	22	345	366	UUUGCAGUUCGAAAGU GGAAGC	:: ::::: :..:::: .	CUCUCGACUUUCCAGU UGCAAG	Translation	1
Hbr-miR9386b	HbLBD32	4	1	22	345	366	UUUGCAGUUCGAAAGU GGAAGC	:: ::::: :..:::: .	CUCUCGACUUUCCAGU UGCAAG	Translation	1
Hbr-miRN1507	HbLBD40	4	1	21	755	775	AAUGGGUAGGUGGGUA AGAUG	: .::.::.:: :::.. :::	CUUUUUGCCUACAUAC UUAUU	Cleavage	1
Hbr-miRN1509	HbLBD18	4	1	21	3560	3580	CAGUUGAUGCAUUGUA UGAUU	::: ::::::::: ::: :	CAUCUUACAAUGCAUG AACAG	Cleavage	1

Hbr-miRN1513	HbLBD18	4	1	21	4459	4479	UGUUGGAAGUCUUUGA AGGGG	: :.:: :::::.::. : :::	CACUUUGAAAGAUUUU GAACA	Cleavage	1
Hbr-miRN1519	HbLBD21	4	1	21	628	648	UUGGCAGCAAGGACUU UGGGU	: ::::::..::: :::: :.	UCACAAAGUUUUUGGU GCCAG	Cleavage	1
Hbr-miR156a	HbLBD32	4.5	1	21	1598	1618	UGACAGAAGAGAGAGA GCACA	: .:::.:.::::.:::.	AUUUUUCUUUUUCUUU UGUUU	Cleavage	1
Hbr-miR156a	HbLBD15	4.5	1	21	28	48	UGACAGAAGAGAGAGA GCACA	: .:::::.:::::: ::	CCUCUUCUCUUUCUUC UCUCU	Cleavage	1
Hbr-miR159b	HbLBD25	4.5	1	21	354	374	AUCUGCCGACUCAUUC AUUCA	:.::: :::.:::::..:.	CCAGUGUAUGGGUCGG UGGGG	Cleavage	1
Hbr-miR159c	HbLBD11	4.5	1	20	524	543	AUUGGAGUGAAGGGAG CUCU	.:: :::: :::::: :.::	GGAACUCCGAUCACUC CGAU	Translation	1
Hbr-miR159d	HbLBD11	4.5	1	20	524	543	AUUGGAGUGAAGGGAG CUCU	.:: :::: :::::: :.::	GGAACUCCGAUCACUC CGAU	Translation	1
Hbr-miR159e	HbLBD11	4.5	1	21	523	543	AUUGGAGUGAAGGGAG CUCGG	:: :::: ::::::: .::	AGGAACUCCGAUCACU CCGAU	Translation	1
Hbr-miR164a	HbLBD21	4.5	1	21	1271	1291	UGGAGAAGCAGGGCAC GUGCA	.:.:: .:.: ::::::: :	GAUAUGUUUCUUUCUU CUCCA	Cleavage	1
Hbr-miR164b	HbLBD21	4.5	1	21	1271	1291	UGGAGAAGCAGGGCAC GUGCA	.:.:: .:.: ::::::: :	GAUAUGUUUCUUUCUU CUCCA	Cleavage	1
Hbr-miR164c	HbLBD21	4.5	1	21	1271	1291	UGGAGAAGCAGGGCAC GUGCA	.:.:: .:.: ::::::: :	GAUAUGUUUCUUUCUU CUCCA	Cleavage	1
Hbr-miR164d	HbLBD21	4.5	1	21	1271	1291	UGGAGAAGCAGGGCAC GUGCA	.:.:: .:.: ::::::: :	GAUAUGUUUCUUUCUU CUCCA	Cleavage	1
Hbr-miR164e	HbLBD39	4.5	1	21	254	273	UGGAGAAGCAGGGCAC AUGCU	: ::: :..:::::::: .	CUCUUGU- CUUUGCUUCUCCG	Cleavage	1
Hbr-miR164f	HbLBD21	4.5	1	21	1271	1291	UGGAGAAGCAGGGCAC GUGCA	.:.:: .:.: ::::::: :	GAUAUGUUUCUUUCUU CUCCA	Cleavage	1
Hbr-miR164g	HbLBD21	4.5	1	21	1271	1291	UGGAGAAGCAGGGCAC GUGCA	.:.:: .:.: ::::::: :	GAUAUGUUUCUUUCUU CUCCA	Cleavage	1
Hbr-miR164h	HbLBD21	4.5	1	21	1271	1291	UGGAGAAGCAGGGCAC GUGCA	.:.:: .:.: ::::::: :	GAUAUGUUUCUUUCUU CUCCA	Cleavage	1
Hbr-miR172a	HbLBD15	4.5	1	21	353	372	AGAAUCUUGAUGAUGC UGCAU	:.: :::::::::: ::: :	CAGUAACAUCAUCAAG- UUCU	Cleavage	1

Hbr-miR172a	HbLBD17	4.5	1	21	281	300	AGAAUCUUGAUGAUGC ::: ::::::.::: ::: UAGCAACAUCAUUAAG-<br>UGCAU : UUCU	Cleavage	1
Hbr-miR172b	HbLBD19	4.5	1	21	296	315	GGAAUCUUGAUGAUGC ::: :::::::::: ::: UAGCAACAUCAUCAAG-<br>UGCAG . UUCU	Cleavage	1
Hbr-miR172c	HbLBD19	4.5	1	21	296	315	GGAAUCUUGAUGAUGC ::: :::::::::: ::: UAGCAACAUCAUCAAG-<br>UGCAG . UUCU	Cleavage	1
Hbr-miR172d	HbLBD15	4.5	1	21	353	372	AGAAUCUUGAUGAUGC :.: :::::::::: ::: CAGUAACAUCAUCAAG-<br>UGCAU : UUCU	Cleavage	1
Hbr-miR172d	HbLBD17	4.5	1	21	281	300	AGAAUCUUGAUGAUGC ::: ::::::.::: ::: UAGCAACAUCAUUAAG-<br>UGCAU : UUCU	Cleavage	1
Hbr-miR172e	HbLBD15	4.5	1	21	353	372	AGAAUCUUGAUGAUGC :.: :::::::::: ::: CAGUAACAUCAUCAAG-<br>UGCAU : UUCU	Cleavage	1
Hbr-miR172e	HbLBD17	4.5	1	21	281	300	AGAAUCUUGAUGAUGC ::: ::::::.::: ::: UAGCAACAUCAUUAAG-<br>UGCAU : UUCU	Cleavage	1
Hbr-miR395e	HbLBD42	4.5	1	21	941	961	CUGAAGGGUUUGGAGG ::: :.:..:: :::::. CAGUACUUUUAACCCC<br>AACUC :: UUUAG	Cleavage	1
Hbr-miR395f	HbLBD42	4.5	1	21	941	961	CUGAAGGGUUUGGAGG ::: :.:..:: :::::. CAGUACUUUUAACCCC<br>AACUC :: UUUAG	Cleavage	1
Hbr-miR396a	HbLBD38	4.5	1	21	620	640	UUCCACAGCUUUCUUG :.: :.:.::.:::::: AGGGUUAGGAGAGCUG<br>AACUU :. UGAAG	Cleavage	1
Hbr-miR396b	HbLBD38	4.5	1	21	620	640	UUCCACAGCUUUCUUG .: :.:.::.:::::: AGGGUUAGGAGAGCUG<br>AACUG :. UGAAG	Cleavage	1
Hbr-miR396c	HbLBD38	4.5	1	21	620	640	UUCCACAGCUUUCUUG :.: :.:.::.:::::: AGGGUUAGGAGAGCUG<br>AACUU :. UGAAG	Cleavage	1
Hbr-miR396d	HbLBD38	4.5	1	21	620	640	UUCCACAGCUUUCUUG .: :.:.::.:::::: AGGGUUAGGAGAGCUG<br>AACUG :. UGAAG	Cleavage	1
Hbr-miR396e	HbLBD38	4.5	1	21	620	640	UUCCACAGCUUUCUUG :.: :.:.::.:::::: AGGGUUAGGAGAGCUG<br>AACUU :. UGAAG	Cleavage	1
Hbr-miR397a	HbLBD35	4.5	1	21	403	423	UCAUUGAGUGCAGCGU ::.:: .::::: ..:: UCUCGACUUUGCACUA<br>UGAUG : GGUGA	Cleavage	1
Hbr-miR397b	HbLBD35	4.5	1	21	403	423	UCAUUGAGUGCAGCGU ::.:: .::::: ..:: UCUCGACUUUGCACUA<br>UGAUG : GGUGA	Cleavage	1
Hbr-miR399d	HbLBD56	4.5	1	20	1570	1589	UGCCAAAGGAGAAUUG : : :.::::.:::: :: UGAGAAGUUCUUCUUU<br>CCCU : UGCA	Cleavage	1

Hbr-miR399e	HbLBD56	4.5	1	20	1570	1589	UGCCAAAGGAGAAUUG CCCU	: : :.::::.:::: :: :	UGAGAAGUUCUUCUUU UGCA	Cleavage	1
Hbr-miR6171	HbLBD43	4.5	1	21	376	396	AUGUGGAUUGCUGAAG GCUUU	.:: :::::::.: :::	GCCGUCCUCAGCAAUU CUCAU	Cleavage	1
Hbr-miRN1497	HbLBD6	4.5	1	21	512	532	UAUGGUGUGUUACUUU UGAGU	.:: :: ::::..: ::: ::	CUUCCAAUGUAAUGCC CCAUA	Cleavage	1
Hbr-miRN1500	HbLBD4	4.5	1	21	1627	1647	UGAAGAUAAGAGUCUG UUUGA	::: .::.:.::::.:: :	CGAAAAGGAUUUUUAU UUUGA	Cleavage	1
Hbr-miRN1513	HbLBD53	4.5	1	20	1	20	UGUUGGAAGUCUUUGA AGGG	:::.::::::::.:: :	GGCUUUAAAGACUUUC AUCU	Cleavage	1
Hbr-miRN1518	HbLBD24	4.5	1	22	597	618	CGCAAGUUGUUUCUUG GCUAUG	.: :.::::.::.:: :: :	AGCGGACGAGAAGCAG CUGGCG	Cleavage	1
Hbr-miRN1519	HbLBD26	4.5	1	21	145	165	UUGGCAGCAAGGACUU UGGGU	: :::.:::.::: :::. :.	UCACAAGGUCUUUGGU GCUAG	Cleavage	1
Hbr-miRN1519	HbLBD3	4.5	1	21	363	383	UUGGCAGCAAGGACUU UGGGU	: :::.:::.::: :::. :.	CCACAAGGUCUUUGGU GCUAG	Cleavage	1
Hbr-miRN1519	HbLBD16	4.5	1	21	430	450	UUGGCAGCAAGGACUU UGGGU	: .:.::::.::: :::: :.	UCAUAGAGUCUUUGGU GCCAG	Cleavage	1
Hbr-miRN1519	HbLBD51	4.5	1	21	680	700	UUGGCAGCAAGGACUU UGGGU	: ::::::..::: :::. :.	UCACAAAGUUUUUGGU GCUAG	Cleavage	1
Hbr-miRN1519	HbLBD38	4.5	1	21	271	291	UUGGCAGCAAGGACUU UGGGU	::: :::.:: .::: :::	CGCCACUGUCUUUCUU GCCAA	Cleavage	1
Hbr-miRN1519	HbLBD13	4.5	1	21	227	247	UUGGCAGCAAGGACUU UGGGU	::: :::.:::. :: :::	CGCCACCGUCUUUGUC GCCAA	Cleavage	1
Jcu-miR172b	JcLBD4	4	1	21	643	663	AGAAUCUUGAUGAUGC UGCAU	..: .:.::::::::::	CAAUGGAGUUAUCAAG AUUCU	Cleavage	1
Jcu-miR172c	JcLBD4	4	1	21	643	663	AGAAUCUUGAUGAUGC UGCAU	..: .:.::::::::::	CAAUGGAGUUAUCAAG AUUCU	Cleavage	1
Jcu-miR172d	JcLBD4	4	1	21	643	663	AGAAUCUUGAUGAUGC UGCAU	..: .:.::::::::::	CAAUGGAGUUAUCAAG AUUCU	Cleavage	1
Jcu-miRN1624	JcLBD16	4	1	20	1169	1188	UCCAAUAAAUUCAACU CGUA	::: ::::. :::::::	CUCGAUUUGAGAUUAU UGGA	Cleavage	1
Jcu-miR156a	JcLBD20	4.5	1	21	277	297	UUGACAGAAGAGAGAG AGCAC	:: .:.:::.::::: : .::	GUCUUUUCUUUCUUCU AUUAA	Cleavage	3

Jcu-miR159	JcLBD5	4.5	1	21	534	554	UUUGGAUUGAAGGGAG CUCUA	:::: :: ::::. ::::	CAGAGCACCAAUCAAU UCAAA	Translation	1
Jcu-miR164a	JcLBD3	4.5	1	21	1827	1847	UGGAGAAGCAGGGCAC GUGCA	: ::. ::::.: .:::: :.:	UCCAUAUGCCUUUUUU CUCUA	Cleavage	1
Jcu-miR164a	JcLBD14	4.5	1	21	1414	1434	UGGAGAAGCAGGGCAC GUGCA	:: : :..:::::: .::	CUCAGAUCCUUUGCUU CUUCA	Cleavage	1
Jcu-miR164b	JcLBD3	4.5	1	21	1827	1847	UGGAGAAGCAGGGCAC GUGCA	: ::. ::::.: .:::: :.:	UCCAUAUGCCUUUUUU CUCUA	Cleavage	1
Jcu-miR164b	JcLBD14	4.5	1	21	1414	1434	UGGAGAAGCAGGGCAC GUGCA	:: : :..:::::: .::	CUCAGAUCCUUUGCUU CUUCA	Cleavage	1
Jcu-miR164c	JcLBD3	4.5	1	21	1827	1847	UGGAGAAGCAGGGCAC GUGCA	: ::. ::::.: .:::: :.:	UCCAUAUGCCUUUUUU CUCUA	Cleavage	1
Jcu-miR164c	JcLBD14	4.5	1	21	1414	1434	UGGAGAAGCAGGGCAC GUGCA	:: : :..:::::: .::	CUCAGAUCCUUUGCUU CUUCA	Cleavage	1
Jcu-miR172a	JcLBD4	4.5	1	21	643	663	GGAAUCUUGAUGAUGC UGCAG	..: .:.:::::::::.	CAAUGGAGUUAUCAAG AUUCU	Cleavage	1
Jcu-miR172b	JcLBD1	4.5	1	21	547	567	AGAAUCUUGAUGAUGC UGCAU	::::::::::.::::	GGUAUUCAUCAUCAAG GUUCU	Cleavage	1
Jcu-miR172b	JcLBD22	4.5	1	21	2	21	AGAAUCUUGAUGAUGC UGCAU	::: :.:::::: :::: :	UGGCAUCGUCAUCAA- AUUCU	Cleavage	1
Jcu-miR172c	JcLBD1	4.5	1	21	547	567	AGAAUCUUGAUGAUGC UGCAU	::::::::::.::::	GGUAUUCAUCAUCAAG GUUCU	Cleavage	1
Jcu-miR172c	JcLBD22	4.5	1	21	2	21	AGAAUCUUGAUGAUGC UGCAU	::: :.:::::: :::: :	UGGCAUCGUCAUCAA- AUUCU	Cleavage	1
Jcu-miR172d	JcLBD1	4.5	1	21	547	567	AGAAUCUUGAUGAUGC UGCAU	::::::::::.::::	GGUAUUCAUCAUCAAG GUUCU	Cleavage	1
Jcu-miR172d	JcLBD22	4.5	1	21	2	21	AGAAUCUUGAUGAUGC UGCAU	::: :.:::::: :::: :	UGGCAUCGUCAUCAA- AUUCU	Cleavage	1
Jcu-miR395a	JcLBD15	4.5	1	21	418	438	CUGAAGAGUUUGGAGG AACUC	.:: ::: ::::.:::: ::	CGGUGCCUGCAAAUUC UUGAG	Cleavage	1
Mes-miR172a	MeLBD6	3	1	21	269	289	AGAAUCUUGAUGAUGC UGCAU	: ::: ::::::.::: : :::	AAGCAACAUCAUUAAG UUUCU	Cleavage	1
Mes-miR172b	MeLBD6	3	1	21	269	289	AGAAUCUUGAUGAUGC UGCAU	: ::: ::::::.::: : :::	AAGCAACAUCAUUAAG UUUCU	Cleavage	1

Mes-miR172c	MeLBD6	3	1	21	269	289	AGAAUCUUGAUGAUGCUGCAU	: ::: ::::::.::: : :::	AAGCAACAUCAUUAAGUUUCU	Cleavage	1
Mes-miR171h	MeLBD51	3.5	1	21	121	141	UUGAGCCGCGUCAAUAUCUCC	: :::: :::: :::::: ::	GCAGAUCUUGAAGCGGCUCAU	Translation	1
Mes-miR171k	MeLBD51	3.5	1	21	121	141	UUGAGCCGCGUCAAUAUCUCU	:::: :::: ::::::::	GCAGAUCUUGAAGCGGCUCAU	Translation	1
Mes-miR172d	MeLBD6	3.5	1	21	269	289	GGAAUCUUGAUGAUGCUGCAG	::: ::::::.::: ::: .	AAGCAACAUCAUUAAGUUUCU	Cleavage	1
Mes-miR172e	MeLBD6	3.5	1	21	269	289	GGAAUCUUGAUGAUGCUGCAG	::: ::::::.::: ::: .	AAGCAACAUCAUUAAGUUUCU	Cleavage	1
Mes-miR1446a	MeLBD37	4	1	22	119	140	UGAACUCUCUCCCUCAUAGGCU	.:.: ::::::: :::::..	GGUCGCCGAGGGAGAGAGUUUG	Cleavage	1
Mes-miR156b	MeLBD40	4	1	21	58	78	UUGACAGAAGAGAGAGAGCAC	::::::.:::.::.: :. :.	CUGCUCUUUCUUUUUUUUUAG	Cleavage	1
Mes-miR172a	MeLBD30	4	1	21	296	315	AGAAUCUUGAUGAUGCUGCAU	::: :::::::::: ::: :	CAGCAACAUCAUCAAG-UUCU	Cleavage	1
Mes-miR172b	MeLBD30	4	1	21	296	315	AGAAUCUUGAUGAUGCUGCAU	::: :::::::::: ::: :	CAGCAACAUCAUCAAG-UUCU	Cleavage	1
Mes-miR172c	MeLBD30	4	1	21	296	315	AGAAUCUUGAUGAUGCUGCAU	::: :::::::::: ::: :	CAGCAACAUCAUCAAG-UUCU	Cleavage	1
Mes-miRN1870	MeLBD26	4	1	21	91	111	UGGGUUGUAGAAAGGGGUAAU	: .:.::::::.::::.:	CCUCUCUCUUUCUGCAACUCC	Cleavage	1
Mes-miRN1860	MeLBD15	4	1	22	19	41	UAG-GGAUGGAGAAUGAAUGGUA	:::::: :::::::::: :::	CUCCAUUCCUUCUCCAUCCACUA	Cleavage	1
Mes-miR1446a	MeLBD2	4.5	1	22	85	106	UGAACUCUCUCCCUCAUAGGCU	:::.:::::.:.:::..	GCUAAAUGGGGGAGGGGGUUUG	Cleavage	1
Mes-miR1446b	MeLBD37	4.5	1	22	119	140	UGAACUCUCCCCCUCAACGGCU	.:.:: ::::: ::: :::::..	GGUCGCCGAGGGAGAGAGUUUG	Translation	1
Mes-miR1446c	MeLBD37	4.5	1	22	119	140	UGAACUCUCCCCCUCAACGGCU	.:.:: ::::: ::: :::::..	GGUCGCCGAGGGAGAGAGUUUG	Translation	1
Mes-miR156b	MeLBD3	4.5	1	21	814	834	UUGACAGAAGAGAGAGAGCAC	: .:.:.:.:.::::::. .:	UUUUUUUUUUUUUUCUGUUGA	Cleavage	1

Mes-miR156c	MeLBD40	4.5	1	20	58	77	UGACAGAAGAGAGUGAGCAC	::::: .:::.::.: :. :	CUGCUCUUUCUUUUUUUUUA	Cleavage	1
Mes-miR156d	MeLBD40	4.5	1	20	58	77	UGACAGAAGAGAGUGAGCAC	::::: .:::.::.: :. :	CUGCUCUUUCUUUUUUUUUA	Cleavage	1
Mes-miR156e	MeLBD40	4.5	1	20	58	77	UGACAGAAGAGAGUGAGCAC	::::: .:::.::.: :. :	CUGCUCUUUCUUUUUUUUUA	Cleavage	1
Mes-miR156f	MeLBD40	4.5	1	20	58	77	UGACAGAAGAGAGUGAGCAC	::::: .:::.::.: :. :	CUGCUCUUUCUUUUUUUUUA	Cleavage	1
Mes-miR156g	MeLBD40	4.5	1	20	58	77	UGACAGAAGAGAGUGAGCAC	::::: .:::.::.: :. :	CUGCUCUUUCUUUUUUUUUA	Cleavage	1
Mes-miR156h	MeLBD40	4.5	1	20	58	77	UGACAGAAGAGAGUGAGCAC	::::: .:::.::.: :. :	CUGCUCUUUCUUUUUUUUUA	Cleavage	1
Mes-miR156i	MeLBD40	4.5	1	20	58	77	UGACAGAAGAGAGUGAGCAC	::::: .:::.::.: :. :	CUGCUCUUUCUUUUUUUUUA	Cleavage	1
Mes-miR157a	MeLBD42	4.5	1	21	549	569	UGAUAGAAGAUAGCGAGCACA	: ::: : ::::.::: : ::.	UUUGCACCCUAUUUUCCAUCG	Cleavage	1
Mes-miR172a	MeLBD18	4.5	1	21	382	401	AGAAUCUUGAUGAUGCUGCAU	:.: ::::::::: ::: :	CAGUAACAUCAUCAAG-UUCU	Cleavage	1
Mes-miR172b	MeLBD18	4.5	1	21	382	401	AGAAUCUUGAUGAUGCUGCAU	:.: ::::::::: ::: :	CAGUAACAUCAUCAAG-UUCU	Cleavage	1
Mes-miR172c	MeLBD18	4.5	1	21	382	401	AGAAUCUUGAUGAUGCUGCAU	:.: ::::::::: ::: :	CAGUAACAUCAUCAAG-UUCU	Cleavage	1
Mes-miR172d	MeLBD30	4.5	1	21	296	315	GGAAUCUUGAUGAUGCUGCAG	: ::: ::::::::: : ::.	CAGCAACAUCAUCAAG-UUCU	Cleavage	1
Mes-miR172d	MeLBD34	4.5	1	21	680	700	GGAAUCUUGAUGAUGCUGCAG	..: :.::::::::: ::	CGAUGGACUUAUCAAGAUUCC	Cleavage	1
Mes-miR172e	MeLBD30	4.5	1	21	296	315	GGAAUCUUGAUGAUGCUGCAG	: ::: ::::::::: : ::.	CAGCAACAUCAUCAAG-UUCU	Cleavage	1
Mes-miR172e	MeLBD34	4.5	1	21	680	700	GGAAUCUUGAUGAUGCUGCAG	..: :.::::::::: ::	CGAUGGACUUAUCAAGAUUCC	Cleavage	1
Mes-miR394a	MeLBD20	4.5	1	20	84	103	UUGGCAUUCUGUCCACCUCC	:::::::.:::: :: ::	UAAGGUGGAUAGAAAGCGAA	Cleavage	1
Mes-miR394b	MeLBD20	4.5	1	20	84	103	UUGGCAUUCUGUCCACCUCC	:::::::.:::: :: ::	UAAGGUGGAUAGAAAGCGAA	Cleavage	1

Mes-miR394c	MeLBD20	4.5	1	20	84	103	UUGGCAUUCUGUCCAC CUCC	:::::::.:::: :: ::	UAAGGUGGAUAGAAAG CGAA	Cleavage	1
Mes-miR396a	MeLBD4	4.5	1	21	610	630	UUCCACAGCUUUCUUG AACUU	.: :::.::.::.:::: :.	CGGGUCAGGAGAGUUG UGAAG	Cleavage	1
Mes-miR396b	MeLBD4	4.5	1	21	610	630	UUCCACAGCUUUCUUG AACUG	:.: :::.::.::.:::: :.	CGGGUCAGGAGAGUUG UGAAG	Cleavage	1
Mes-miR396c	MeLBD4	4.5	1	21	610	630	UUCCACAGCUUUCUUG AACUU	.: :::.::.::.:::: :.	CGGGUCAGGAGAGUUG UGAAG	Cleavage	1
Mes-miR396d	MeLBD4	4.5	1	21	610	630	UUCCACAGCUUUCUUG AACUU	.: :::.::.::.:::: :.	CGGGUCAGGAGAGUUG UGAAG	Cleavage	1
Mes-miR396e	MeLBD4	4.5	1	21	610	630	UUCCACAGCUUUCUUG AACUG	:.: :::.::.::.:::: :.	CGGGUCAGGAGAGUUG UGAAG	Cleavage	1
Mes-miR396f	MeLBD4	4.5	1	21	610	630	UUCCACAGCUUUCUUG AACUU	.: :::.::.::.:::: :.	CGGGUCAGGAGAGUUG UGAAG	Cleavage	1
Mes-miR535c	MeLBD17	4.5	1	21	66	86	UUGACGACGAGAGAGA GCACG	: : ::.:.:::.::. : :::	CUUUCUUUUUCUUGUU AUCAA	Cleavage	1
Mes-miR535d	MeLBD17	4.5	1	21	66	86	UUGACGACGAGAGAGA GCACA	: ::.:.:::.::. ::: :	CUUUCUUUUUCUUGUU AUCAA	Cleavage	1
Mes-miRN1836	MeLBD12	4.5	1	21	413	433	UAUGGGGGGAUUGGGC AAAAU	::: :::::::: :..:	CGUUUCCCCAAUCCCAC UGUU	Cleavage	1
Mes-miRN1837a	MeLBD29	4.5	1	21	852	872	UGAAGUGAGUAGAUUU GGUUA	::.: :::.::::::::	GAUACAGAACUAUUCA CUUCA	Cleavage	1
Mes-miRN1837b	MeLBD29	4.5	1	21	852	872	UGAAGUGAGUAGAUUU GGUUA	::.: :::.::::::::	GAUACAGAACUAUUCA CUUCA	Cleavage	1
Mes-miRN1837c	MeLBD29	4.5	1	21	852	872	UGAAGUGAGUAGAUUU GGUUA	::.: :::.::::::::	GAUACAGAACUAUUCA CUUCA	Cleavage	1
Mes-miRN1860	MeLBD45	4.5	1	22	23	45	UAG-GGAUGGAGAAUGAAUG GUA	:::::. ::::::::: :::	CUCCAUUUCUUCUCCA UCCACUA	Cleavage	1
Mes-miRN1866a	MeLBD36	4.5	1	22	1603	1624	CAGGUUGUAGAUAACG UAGGCA	.::.: :: :.::::.:. ::	GCUCUGCAUUUUUUAC AGCUUG	Cleavage	1
Mes-miRN1866b	MeLBD36	4.5	1	22	1603	1624	CAGGUUGUAGAUAACG UAGGCA	.::.: :: :.::::.:. ::	GCUCUGCAUUUUUUAC AGCUUG	Cleavage	1

Mes-<br>miRN1866c	MeLBD36	4.5	1	22	1603	1624	CAGGUUGUAGAUAACG<br>UAGGCA	.::.: :: :.::::.:.<br>::	GCUCUGCAUUUUUUAC<br>AGCUUG	Cleavage	1
Mes-<br>miRN1866d	MeLBD36	4.5	1	22	1603	1624	CAGGUUGUAGAUAACG<br>UAGGCA	.::.: :: :.::::.:.<br>::	GCUCUGCAUUUUUUAC<br>AGCUUG	Cleavage	1
Mes-<br>miRN1866e	MeLBD36	4.5	1	22	1603	1624	CAGGUUGUAGAUAACG<br>UAGGCA	.::.: :: :.::::.:.<br>::	GCUCUGCAUUUUUUAC<br>AGCUUG	Cleavage	1
Mes-<br>miRN1866g	MeLBD36	4.5	1	22	1603	1624	CAGGUUGUAGAUAACG<br>UAGGCA	.::.: :: :.::::.:.<br>::	GCUCUGCAUUUUUUAC<br>AGCUUG	Cleavage	1
Mes-<br>miRN1866h	MeLBD36	4.5	1	22	1603	1624	CAGGUUGUAGAUAACG<br>UAGGCA	.::.: :: :.::::.:.<br>::	GCUCUGCAUUUUUUAC<br>AGCUUG	Cleavage	1
Mes-miRN1875	MeLBD20	4.5	1	22	463	484	AAGUAGAAUUUGCCAU<br>AGACGU	: :: ::::::::<br>:::	CGAUAUACUGCAAAUU<br>CUUCUU	Cleavage	1
Mes-miRN1878	MeLBD36	4.5	1	22	1418	1439	CAAGGAUAUAGGUUUA<br>UGCAUA	: ::::..:::: :.<br>:::	GAAGCAUAGGCCUAUC<br>UUGUUG	Cleavage	1
Rco-miR156e	RcLBD7	4	1	20	583	602	UGACAGAAGAGAGAGA<br>GCAC	::.:::: : .:::::::<br>:	CUGUUCUCCCCUUUCU<br>GUCA	Translation	2
Rco-miR164d	RcLBD20	4	1	21	1296	1316	UGGAGAAGCAGGGCAC<br>AUGCU	.:::: .:.: ::::::<br>:	GAUAUGUUUCUUUCUU<br>CUCCA	Cleavage	1
Rco-miR156e	RcLBD7	4.5	1	20	393	412	UGACAGAAGAGAGAGA<br>GCAC	: .:::.:.:::::: .<br>:	UUCUUCUUUUUCUUCU<br>GCUA	Cleavage	2
Rco-miR156e	RcLBD20	4.5	1	20	259	278	UGACAGAAGAGAGAGA<br>GCAC	: ::.:::.::::.: :.<br>:	AUUCUUUCUUUCUUUU<br>AUUA	Cleavage	2
Rco-miR157a	RcLBD11	4.5	1	21	1581	1601	UUGACAGAAGAUAGAG<br>AGCAC	:.:::.:.::::::..:	UGAAUUUCUGUUUUCU<br>GUUGA	Cleavage	1
Rco-miR157b	RcLBD11	4.5	1	21	1581	1601	UUGACAGAAGAUAGAG<br>AGCAC	:.:::.:.::::::..:	UGAAUUUCUGUUUUCU<br>GUUGA	Cleavage	1
Rco-miR157c	RcLBD11	4.5	1	21	1581	1601	UUGACAGAAGAUAGAG<br>AGCAC	:.:::.:.::::::..:	UGAAUUUCUGUUUUCU<br>GUUGA	Cleavage	1
Rco-miR164a	RcLBD20	4.5	1	21	1296	1316	UGGAGAAGCAGGGCAC<br>GUGCA	.:.:: .:.: ::::::<br>:	GAUAUGUUUCUUUCUU<br>CUCCA	Cleavage	1
Rco-miR164b	RcLBD20	4.5	1	21	1296	1316	UGGAGAAGCAGGGCAC<br>GUGCA	.:.:: .:.: ::::::<br>:	GAUAUGUUUCUUUCUU<br>CUCCA	Cleavage	1
Rco-miR164c	RcLBD20	4.5	1	21	1296	1316	UGGAGAAGCAGGGCAC<br>GUGCA	.:.:: .:.: ::::::<br>:	GAUAUGUUUCUUUCUU<br>CUCCA	Cleavage	1

Rco-miR172b	RcLBD24	4.5	1	21	226	245	AGAAUCUUGAUGAUGC UGCAU	::. :::::::: :::: :	UGGCGUCAUCAUCAA-AUUCU	Cleavage	1
Rco-miR172c	RcLBD24	4.5	1	21	226	245	AGAAUCUUGAUGAUGC UGCAU	::. :::::::: :::: :	UGGCGUCAUCAUCAA-AUUCU	Cleavage	1
Rco-miR172d	RcLBD24	4.5	1	21	226	245	AGAAUCUUGAUGAUGC UGCAU	::. :::::::: :::: :	UGGCGUCAUCAUCAA-AUUCU	Cleavage	1
Rco-miR393	RcLBD9	4.5	1	21	568	588	UCCAAAGGGAUCGCAU UGAUC	.:.:::: . :.:::: ::	AGUUAAUGCCGACUCU UUGGA	Translation	1
Rco-miRN2947	RcLBD1	4.5	1	21	287	307	UAUGGGAGGCAUGGUC AGAAA	::: ::.:..::.:::: : :	UUUAUGGCUGUGUCUC CCACA	Cleavage	1

Table S3. Duplication analysis of *LBD* genes in Euphorbiaceae

Species	Gene Type	Genes
Jatropha curcas	Singleton gene	None
	Dispersed duplication gene	JcLBD26,JcLBD30,JcLBD2,JcLBD3,JcLBD4,JcLBD11,JcLBD12,JcLBD13,JcLBD17, JcLBD18,JcLBD19,JcLBD22,JcLBD25
	Proximal duplication gene	None
	Tandem duplication gene	JcLBD6,JcLBD7,JcLBD8,JcLBD14,JcLBD15
	Segmental duplication gene	JcLBD1,JcLBD27,JcLBD28,JcLBD29,JcLBD31,JcLBD32,JcLBD33,JcLBD5,JcLBD9, JcLBD10,JcLBD16,JcLBD20,JcLBD21,JcLBD23,JcLBD24
Hevea brasiliensis	Singleton gene	None
	Dispersed duplication gene	HbLBD13,HbLBD15,HbLBD16,HbLBD17,HbLBD18,HbLBD19,HbLBD20,HbLBD22, HbLBD3,HbLBD23,HbLBD24,HbLBD25,HbLBD26,HbLBD27,HbLBD28,HbLBD29, HbLBD30,HbLBD31,HbLBD32,HbLBD33,HbLBD34,HbLBD35,HbLBD36,HbLBD37, HbLBD39,HbLBD41,HbLBD42,HbLBD5,HbLBD43,HbLBD46,HbLBD47,HbLBD48, HbLBD49,HbLBD50,HbLBD52,HbLBD55,HbLBD58,HbLBD8
	Proximal duplication gene	HbLBD44,HbLBD45
	Tandem duplication gene	HbLBD54,HbLBD6,HbLBD7,HbLBD56,HbLBD57,HbLBD10
	Segmental duplication gene	HbLBD1,HbLBD2,HbLBD14,HbLBD21,HbLBD4,HbLBD38,HbLBD40,HbLBD51, HbLBD53,HbLBD9,HbLBD11,HbLBD12
Manihot esculenta	Singleton gene	None
	Dispersed duplication gene	MeLBD33,MeLBD44,MeLBD49,MeLBD54,MeLBD10,MeLBD12,MeLBD17,MeLBD24
	Proximal duplication gene	None
	Tandem duplication gene	MeLBD3,MeLBD37,MeLBD42,MeLBD9
	Segmental duplication gene	MeLBD1,MeLBD2,MeLBD26,MeLBD27,MeLBD28,MeLBD29,MeLBD30,MeLBD31, MeLBD32,MeLBD34,MeLBD35,MeLBD36,MeLBD38,MeLBD39,MeLBD40,MeLBD41,

		MeLBD43,MeLBD45,MeLBD46,MeLBD47,MeLBD48,MeLBD50,MeLBD51,MeLBD52, MeLBD53,MeLBD4,MeLBD5,MeLBD6,MeLBD7,MeLBD8,MeLBD11,MeLBD13, MeLBD14,MeLBD15,MeLBD16,MeLBD18,MeLBD19,MeLBD20,MeLBD21,MeLBD22, MeLBD23,MeLBD25
Ricinus communis	Singleton gene	None
	Dispersed duplication gene	RcLBD1,RcLBD10,RcLBD11,RcLBD14,RcLBD16,RcLBD17,RcLBD18,RcLBD19, RcLBD20,RcLBD2,RcLBD21,RcLBD24,RcLBD25,RcLBD28,RcLBD3,RcLBD4,RcLBD5, RcLBD6,RcLBD7,RcLBD9
	Proximal duplication gene	RcLBD26,RcLBD27
	Tandem duplication gene	RcLBD23
	Segmental duplication gene	RcLBD12,RcLBD13,RcLBD15,RcLBD22,RcLBD29,RcLBD30,RcLBD8

Table S4. Analysis of selection pressure on *LBD* duplication gene pairs in Euphorbiaceae

Duplicated Gene Pairs	Duplication Type	Ka	Ks	Ka/Ks	P-Value (Fisher)
HbLBD53-HbLBD54	Tandem duplication	0.279579	2.94822	0.09483	0
HbLBD56-HbLBD57	Tandem duplication	0.164051	0.84081	0.195111	2.13421e-39
HbLBD9-HbLBD10	Tandem duplication	0.335085	2.95259	0.113489	0
HbLBD14-HbLBD4	Segmental duplication	0.079526	0.59612	0.133405	1.60313e-28
HbLBD1-HbLBD2	Segmental duplication	0.002334	0.009725	0.240031	0.142303
HbLBD21-HbLBD51	Segmental duplication	0.041734	0.256986	0.162397	4.2452e-12
HbLBD38-HbLBD11	Segmental duplication	0.06326	0.303998	0.208093	4.40616e-16
HbLBD40-HbLBD12	Segmental duplication	0.021696	0.173796	0.124836	7.08127e-07
HbLBD53-HbLBD9	Segmental duplication	0.332146	2.76695	0.120041	0
JcLBD14-JcLBD15	Tandem duplication	0.328406	3.11877	0.1053	0
JcLBD6-JcLBD7	Tandem duplication	0.073491	0.327119	0.224662	4.04604e-14
JcLBD7-JcLBD8	Tandem duplication	0.204599	1.35522	0.15097	3.75782e-63
JcLBD16-JcLBD23	Segmental duplication	0.484164	2.38805	0.202745	0
JcLBD1-JcLBD21	Segmental duplication	0.52553	2.98619	0.175987	0
JcLBD20-JcLBD24	Segmental duplication	0.160243	1.21961	0.131389	2.973e-52
JcLBD27-JcLBD32	Segmental duplication	0.208852	3.12213	0.066894	0
JcLBD28-JcLBD33	Segmental duplication	0.451834	3.27046	0.138156	0
JcLBD29-JcLBD10	Segmental duplication	0.28411	3.54093	0.080236	0
JcLBD31-JcLBD9	Segmental duplication	0.650161	2.60271	0.249801	0
JcLBD5-JcLBD16	Segmental duplication	0.351508	2.66302	0.131996	0
JcLBD5-JcLBD23	Segmental duplication	0.290298	2.84607	0.102	0
MeLBD2-MeLBD3	Tandem duplication	0.350882	2.693	0.130294	0
MeLBD36-MeLBD37	Tandem duplication	0.185011	0.917502	0.201647	2.14285e-42

MeLBD41-MeLBD42	Tandem duplication	0.186264	1.0994	0.169424	2.23217e-50
MeLBD8-MeLBD9	Tandem duplication	0.334432	2.74392	0.121881	0
MeLBD1-MeLBD13	Segmental duplication	0.347697	3.13173	0.111024	0
MeLBD1-MeLBD47	Segmental duplication	0.339879	3.23424	0.105088	0
MeLBD1-MeLBD4	Segmental duplication	0.061464	0.389418	0.157834	1.5847e-23
MeLBD23-MeLBD25	Segmental duplication	0.06301	0.473301	0.133128	3.91304e-23
MeLBD26-MeLBD22	Segmental duplication	0.02981	0.403271	0.073921	1.54937e-23
MeLBD27-MeLBD21	Segmental duplication	0.037453	0.578759	0.064712	2.28038e-25
MeLBD28-MeLBD20	Segmental duplication	0.069934	0.489103	0.142985	7.6212e-18
MeLBD29-MeLBD19	Segmental duplication	0.103573	0.266152	0.389151	1.20651e-05
MeLBD2-MeLBD8	Segmental duplication	0.055743	0.361954	0.154006	5.3287e-19
MeLBD30-MeLBD18	Segmental duplication	0.059983	0.36857	0.162746	1.66621e-15
MeLBD30-MeLBD32	Segmental duplication	0.204969	3.68756	0.055584	0
MeLBD30-MeLBD6	Segmental duplication	0.216842	3.4184	0.063434	0
MeLBD31-MeLBD50	Segmental duplication	0.602035	2.66191	0.226167	0
MeLBD31-MeLBD5	Segmental duplication	0.648078	2.429	0.266808	0
MeLBD31-MeLBD7	Segmental duplication	0.060979	0.507059	0.12026	6.24088e-24
MeLBD32-MeLBD6	Segmental duplication	0.041121	0.460541	0.089289	1.61878e-22
MeLBD34-MeLBD39	Segmental duplication	0.05259	0.397186	0.132407	2.6043e-18
MeLBD35-MeLBD51	Segmental duplication	0.755237	1.98502	0.380469	0
MeLBD36-MeLBD41	Segmental duplication	0.069385	0.422675	0.164157	2.02315e-24
MeLBD38-MeLBD11	Segmental duplication	0.3228	2.86693	0.112594	0
MeLBD38-MeLBD40	Segmental duplication	0.08014	0.451071	0.177666	1.52531e-22
MeLBD40-MeLBD11	Segmental duplication	0.31644	2.861	0.110605	0
MeLBD40-MeLBD53	Segmental duplication	0.294439	2.78404	0.10576	0
MeLBD43-MeLBD16	Segmental duplication	0.031749	0.359071	0.08842	3.5517e-14

MeLBD45-MeLBD15	Segmental duplication	0.039973	0.216213	0.184878	1.46859e-08
MeLBD46-MeLBD14	Segmental duplication	0.060277	0.739059	0.081559	1.91237e-46
MeLBD47-MeLBD13	Segmental duplication	0.073219	0.402579	0.181876	1.15235e-18
MeLBD47-MeLBD4	Segmental duplication	0.306145	3.35408	0.091275	0
MeLBD4-MeLBD13	Segmental duplication	0.284267	3.2709	0.086908	0
MeLBD50-MeLBD5	Segmental duplication	0.308693	0.800934	0.385416	1.35691e-13
MeLBD50-MeLBD7	Segmental duplication	0.546632	2.77144	0.197237	0
MeLBD53-MeLBD11	Segmental duplication	0.083221	0.474362	0.175438	1.27406e-19
MeLBD5-MeLBD7	Segmental duplication	0.63584	2.45352	0.259154	0
MeLBD6-MeLBD18	Segmental duplication	0.169242	3.65774	0.046269	0
RcLBD12-RcLBD30	Segmental duplication	0.338409	2.994	0.113029	0
RcLBD13-RcLBD15	Segmental duplication	0.692902	2.37799	0.291381	0
RcLBD22-RcLBD29	Segmental duplication	0.499424	3.05805	0.163314	0
RcLBD30-RcLBD8	Segmental duplication	0.458206	2.56099	0.178917	0

Table S5. Tissue expression of *JcLBD* genes

Name	Fruit	Male Flower	Female Flower	Leaf	Root	Stem
JcLBD6	0.29225	3.86276	0.164039	0	0	0
JcLBD11	0	5.66631	0	0	0.079879	0
JcLBD7	1.72631	5.24471	0.378196	0	2.84791	0
JcLBD8	18.2269	15.6993	3.30854	8.83294	6.98812	0
JcLBD22	0	0.352506	7.69418	4.91956	2.23093	1.59043
JcLBD32	0.089777	4.316	19.0324	0	5.88014	4.96452
JcLBD27	0.174972	2.71576	33.3546	0.326853	6.51011	1.16865
JcLBD18	6.34127	9.85397	44.9917	3.84218	2.03638	68.6991
JcLBD4	0	0	0	0	4.14449	0
JcLBD17	0	0.258832	0	0	1.72765	0
JcLBD13	0.205338	0.070895	0.030409	0	2.80826	0.214844
JcLBD15	0.13586	6.68585	25.2767	0.579699	4.34791	0.250489
JcLBD3	11.1025	9.36227	8.2325	33.6791	8.0526	4.8812
JcLBD14	15.8558	1.16658	2.87907	0.225872	2.04193	1.13789
JcLBD24	0.064276	0.098973	0	0	43.5276	0.628414
JcLBD20	7.3212	1.87752	0.11304	0.904383	11.2486	4.17
JcLBD19	0	1.67893	6.06907	0	12.1686	0
JcLBD25	0	0	0.049509	0	0.292256	0
JcLBD30	0.202495	2.79672	3.18425	0.292029	10.4211	1.96696
JcLBD26	0.9837	0	0.676357	0	21.5433	6.66681
JcLBD2	13.8558	0.190728	3.85147	1.00013	2.40928	0.601426
JcLBD12	0.839434	0.138324	0.246159	0.337614	0	0
JcLBD9	0	0	0.394471	0	0	0
JcLBD31	0	0	0	0	0	0
JcLBD28	0.668832	3.60872	1.04656	0.972837	2.34478	0.799104
JcLBD33	0	0	0	0	0	0
JcLBD21	0.071081	36.1793	0	0	0	0
JcLBD1	0	37.7278	0	0	0	0
JcLBD10	6.68135	27.7906	29.7518	0.053569	27.4384	2.91876
JcLBD29	24.9641	0.075063	0.070782	0	0.984168	0
JcLBD16	1.13017	2.77142	9.10345	17.5085	20.4708	13.8738
JcLBD23	1.20867	15.1682	5.00961	2.28675	8.07656	19.016
JcLBD5	52.3571	35.4776	91.1157	28.9893	98.6094	200.725

* Expression values were TPM values and normalized by quantile normalization.

Table S6. Expression of *JcLBD* genes in drought treatment

Name	C1dL	C4dL	C7dL	D1dL	D4dL	D7dL	C1dR	C4dR	C7dR	D1dR	D4dR	D7dR
JcLBD17	0	0.8958	0.7161	0	0.5413	0	0	4.2884	0.7979	3.2661	1.0758	0.2618
JcLBD25	0	15.973	11.7492	0	9.816	0	0	112.4841	0	91.2312	0	0
JcLBD27	0	0	0	0	0	1.3855	0.2027	0.3717	0.4856	2.3329	0.9145	1.3858
JcLBD19	0	0	1.2435	0	0	42.2908	0.402	5.911	0.8415	2.9548	0	42.3007
JcLBD3	2.8894	2.2024	1.3143	3.1613	2.7438	4.3043	3.5986	1.9142	1.8244	1.0283	2.7519	2.7471
JcLBD14	1.9378	3.6739	3.3624	4.4083	1.3612	1.0627	2.0709	1.3965	1.6683	1.2139	2.0123	1.6823
JcLBD5	447.1215	243.546	273.549	350.49	60.9477	119.3244	208.538	74.4735	227.0475	95.0095	39.9278	131.8013
JcLBD7	0.2565	0.0232	0	0.0958	0	0.0973	0	0.0352	0.2241	0.0341	2.4522	0.0194
JcLBD18	57.7141	29.899	13.3465	21.2514	7.4393	4.9539	26.2628	41.7866	34.2189	28.6894	43.8267	7.648
JcLBD28	0.3763	0	0.8573	0	0	0.2651	0.8315	0.0375	0.5423	0.024	0.277	0.5258
JcLBD24	0.6275	0	0	0	0.2433	6.8932	20.9744	1.5789	23.9671	5.4197	1.1637	7.3656
JcLBD23	0.4034	0.2391	3.3447	1.0799	1.3955	4.2993	2.7387	2.3932	8.0456	2.1	1.0652	6.0785
JcLBD16	1.8946	0.7689	1.2404	0.292	0.6425	0.4009	1.8957	2.5517	7.9713	3.3439	3.1271	1.7808
JcLBD20	2.2467	1.9502	2.3359	2.2739	3.8347	7.7859	34.1733	17.2097	32.0594	13.4818	28.5998	39.6101
JcLBD26	0	0.8344	0.1877	1.0146	0.118	11.7249	65.5748	286.507	120.5565	220.934	197.681	66.8701
JcLBD32	0	0	0	0	0	0.2826	7.9016	5.3752	8.4551	6.4456	36.6279	3.4909
JcLBD30	0	0	0	0	0	0.3195	3.0752	2.7997	1.8696	3.6639	6.2775	2.2052
JcLBD8	0	0.4489	0	0.4394	0	0.2668	1.116	0.6801	0.584	0	0.2788	0.5293
JcLBD13	0.1261	0.0982	0.0276	0.0519	0.0266	3.6897	6.4057	4.2327	0.39	1.9857	0.4285	5.5048
JcLBD10	0.3068	0.1942	0.4783	1.4596	0.3255	37.1506	129.813	35.3881	10.3751	8.0369	3.3957	40.0158
JcLBD4	0	0	0.7681	0	0	0.475	1.0609	9.2373	1.7951	1.7671	1.9573	2.3435
JcLBD15	1.8233	2.5462	0.2549	0.6243	0.4387	1.2822	2.9005	5.3298	2.66	2.7912	2.6674	4.4833

* Expression values were TPM values and normalized by quantile normalization.

Table S7. Expression of *JcLBD* genes in salt treatment

Name	C2hL	C2dL	C7dL	S2hL	S2dL	S7dL	C2hR	C2dR	C7dR	S2hR	S2dR	S7dR
JcLBD14	2.6065	1.9191	3.2615	3.1972	3.7349	2.3419	1.1126	1.1502	1.5546	1.4495	1.6717	2.1946
JcLBD5	397.268	504.7825	266.9745	249.888	560.1935	215.6055	67.1044	77.1787	221.5705	66.1082	127.2545	148.949
JcLBD18	58.7367	41.6491	13.0281	55.6568	30.6625	20.7038	23.3961	34.6226	33.3885	20.4175	20.5228	21.359
JcLBD3	2.7195	1.5237	1.2926	4.4523	3.7539	2.4303	2.1624	1.9492	1.7799	2.5025	1.7551	2.8502
JcLBD25	2.168	15.5785	11.4697	0	0	0	11.7807	28.4072	0	22.0894	0.8855	0
JcLBD19	0	0	1.2133	0	0	0	29.5429	1.0636	0.8213	1.4777	1.481	0
JcLBD29	0	0	0	0.0282	0	0	6.2857	0.3373	0.1697	0.6736	0.3522	1.5424
JcLBD28	0	0.4489	0.8365	0	0	0	0.2452	0	0.5291	0.7217	1.7726	0.2629
JcLBD23	0.1729	0.7219	3.2652	0.1784	0.661	0.364	2.7916	1.376	7.8505	0.4665	1.5038	4.8543
JcLBD24	0	0	0	0	0.1484	0	3.5206	2.3613	23.3161	4.5652	48.2463	3.0524
JcLBD8	0	0	0	0	0.3588	0	0.2629	0.2461	0.5699	0.5128	1.2847	1.5875
JcLBD16	0.6454	0.7552	1.2372	1.2381	3.7216	0	1.1368	6.8292	7.7767	2.3432	7.1989	6.972
JcLBD12	0.3724	0.0636	0.1233	0.1179	0	0.0481	0.0434	0.1553	0.2379	0.2423	0.1878	0.1921
JcLBD27	0	0	0	0	0	1.1207	0.4312	1.175	0.4736	1.6816	2.8316	0.859
JcLBD13	0	0.1305	0.0269	0	0.2757	0	5.6023	8.1741	0.4023	6.5	17.5775	5.3386
JcLBD10	1.003	0.677	0.3142	1.9505	1.4234	0.075	171.9707	39.5508	10.2299	81.3914	345.5519	27.8747
JcLBD4	0.0259	0	0.7494	0	0	0	2.7081	3.2216	1.7733	1.927	1.9752	4.8283
JcLBD30	0	0	0	0	0	0	2.3649	3.9187	1.8238	2.917	2.3586	4.2203
JcLBD20	4.6592	1.4534	2.2798	4.2348	7.8787	2.1044	29.0017	32.1325	31.2627	33.8866	23.6776	57.7015
JcLBD32	0	0.4785	0	0	0	0	14.4227	14.4009	8.2487	12.3535	10.2739	9.3487
JcLBD26	0.6063	1.6984	0.1832	0	0.6961	1.4558	104.9921	153.577	117.62	184.999	80.3794	62.6996
JcLBD17	0	0	0.6991	0	0	0	0.771	0.715	0.7784	4.0505	0.2564	0.6934
JcLBD15	1.1245	2.1922	0.2489	1.4825	0.7648	0.4855	2.1271	1.0028	2.5952	6.253	4.3094	3.3853

* Expression values were TPM values and normalized by quantile normalization.

Table S8. Target genes for JcLBD transcription factors

Motif	Target Genes
Jcr4S00009.60	jc012138, jc000384, jc010116, jc014050, jc001911, jc021062, jc023030, jc018751, jc014708, jc005542, jc002825, jc007763, jc021106, jc022172, jc001513, jc020192, jc003193, jc005317, jc005639, jc001912, jc003981, jc005937, jc008392, jc014865, jc011680, jc007113, jc024838, jc003564, jc022047, jc010191, jc015342, jc011300, jc015612, jc004630, jc018205, jc012116, jc008665, jc021558, jc016201, jc003764, jc008444, jc025263, jc011386, jc009001, jc005981, jc020924, jc005861, jc013092, jc000771, jc023760, jc014955, jc005335, jc005855, jc022048, jc001018, jc019697, jc024585, jc016899, jc007995, jc025266, jc002195, jc010375, jc005982, jc010187, jc023281, jc001823, jc008639, jc005074, jc016209, jc024875, jc024584, jc009307, jc025639, jc014377, jc025569, jc003999, jc022050, jc001165, jc010024, jc019730, jc006415, jc002823, jc022071, jc017629, jc008845, jc018650, jc016805, jc017664, jc009485, jc002661, jc001113, jc005316, jc014293, jc000746, jc018963, jc010604, jc023373, jc002861, jc021560, jc018695, jc019804, jc019713, jc024549, jc010462, jc019411, jc003121, jc006879, jc020298, jc008723, jc009796, jc025753, jc014119, jc002282, jc015785, jc024941, jc015611, jc016729, jc023978, jc003590, jc013795, jc018100, jc018885, jc021840, jc021566, jc023029, jc008025, jc021105, jc003348, jc002692, jc008048, jc012352, jc004079, jc024331, jc001903, jc001764, jc024337, jc000009, jc010142, jc011678, jc010186, jc013222, jc016213, jc018383, jc015525, jc000411, jc000710, jc004637, jc011387
Jcr4S00803.60	jc025266, jc023373, jc001906, jc001911, jc008665, jc017629, jc018885, jc022071, jc019730, jc002823, jc002825, jc011680, jc001903, jc011678, jc019804, jc025263, jc000746, jc006947
Jcr4S13769.10	jc009307, jc018100, jc001165, jc008025, jc010607, jc024584, jc022071, jc023978, jc020924, jc002661, jc010024, jc004630, jc023373, jc010116, jc014865, jc000746, jc020192, jc005335, jc005542, jc023281, jc004079, jc003193, jc015525, jc014708, jc002282, jc009001, jc019804, jc002195, jc000710, jc023029, jc023030, jc008444, jc022172, jc012504, jc025266, jc025569, jc005316, jc007763, jc014119, jc025263, jc011678, jc000771, jc010604, jc013092, jc012116, jc024426, jc001903, jc002825, jc005317, jc019730, jc025639, jc003999, jc007113, jc001018, jc024337, jc021806, jc012138, jc002823, jc013222, jc002692, jc017664, jc001911, jc008723, jc005074, jc020298, jc017629, jc000384, jc011680, jc018885

Table S9. RNA-seq samples in 6 tissues of *J. curcas*

Name	Tissue	Development stage	SRR	Reference
fruit	Seed endosperm	Immature fruit	SRR5974841	PRJNA399212
male flower	Male flower	Fully open	SRR5974843	PRJNA399212
female flower	Female flower	Fully open	SRR5974844	PRJNA399212
leaf	Leaf	Fully mature	SRR5974846	PRJNA399212
root	Root	Fully expanded	SRR5974855	PRJNA399212
stem	Stem	Fully mature	SRR5974856	PRJNA399212

Table S10. RNA-seq samples under stress of *J. curcas.*

Name	Tissue	Treatment	SRR	Reference
C1dL-1	Leaf	Control	SRR1542241	PRJNA257901
C1dL-2	Leaf	Control	SRR1542253	PRJNA257901
D1dL-1	Leaf	Drought	SRR1542247	PRJNA257901
D1dL-2	Leaf	Drought	SRR1542259	PRJNA257901
C4dL-1	Leaf	Control	SRR1542242	PRJNA257901
C4dL-2	Leaf	Control	SRR1542254	PRJNA257901
D4dL-1	Leaf	Drought	SRR1542248	PRJNA257901
D4dL-2	Leaf	Drought	SRR1542260	PRJNA257901
C7dL-11	Leaf	Control	SRR1542243	PRJNA257901
C7dL-21	Leaf	Control	SRR1542255	PRJNA257901
D7dL-1	Leaf	Drought	SRR1542249	PRJNA257901
D7dL-2	Leaf	Drought	SRR1542261	PRJNA257901
C1dR-1	Root	Control	SRR1542235	PRJNA257901
C1dR-2	Root	Control	SRR1542250	PRJNA257901
D1dR-1	Root	Drought	SRR1542244	PRJNA257901
D1dR-2	Root	Drought	SRR1542256	PRJNA257901
C4dR-1	Root	Control	SRR1542239	PRJNA257901
C4dR-2	Root	Control	SRR1542251	PRJNA257901
D4dR-1	Root	Drought	SRR1542245	PRJNA257901
D4dR-2	Root	Drought	SRR1542257	PRJNA257901
C7dR-11	Root	Control	SRR1542240	PRJNA257901
C7dR-21	Root	Control	SRR1542252	PRJNA257901
D7dR-1	Root	Drought	SRR1542246	PRJNA257901
D7dR-2	Root	Drought	SRR1542258	PRJNA257901
C2hL-1	Leaf	Control	SRR1251568	PRJNA244896
C2hL-2	Leaf	Control	SRR1251963	PRJNA244896
S2hL-1	Leaf	Salt	SRR1251958	PRJNA244896
S2hL-2	Leaf	Salt	SRR1251969	PRJNA244896
C2dL-1	Leaf	Control	SRR1251570	PRJNA244896
C2dL-2	Leaf	Control	SRR1251965	PRJNA244896
S2dL-1	Leaf	Salt	SRR1251960	PRJNA244896
S2dL-2	Leaf	Salt	SRR1251971	PRJNA244896

C7dL-1	Leaf	Control	SRR1251572	PRJNA244896
C7dL-2	Leaf	Control	SRR1251967	PRJNA244896
S7dL-1	Leaf	Salt	SRR1251962	PRJNA244896
S7dL-2	Leaf	Salt	SRR1251973	PRJNA244896
C2hR-1	Root	Control	SRR1251567	PRJNA244896
C2hR-2	Root	Control	SRR1251964	PRJNA244896
S2hR-1	Root	Salt	SRR1251957	PRJNA244896
S2hR-2	Root	Salt	SRR1251970	PRJNA244896
C2dR-1	Root	Control	SRR1251569	PRJNA244896
C2dR-2	Root	Control	SRR1251966	PRJNA244896
S2dR-1	Root	Salt	SRR1251959	PRJNA244896
S2dR-2	Root	Salt	SRR1251972	PRJNA244896
C7dR-1	Root	Control	SRR1251571	PRJNA244896
C7dR-2	Root	Control	SRR1251968	PRJNA244896
S7dR-1	Root	Salt	SRR1251961	PRJNA244896
S7dR-2	Root	Salt	SRR1251974	PRJNA244896
